# *Tagln1-Cre*–driven Ercc1 deficiency reveals sex-specific effects on adipose tissue in mice

**DOI:** 10.1210/endocr/bqag106

**Published:** 2026-09-16

**Authors:** Annika A Jüttner, Patric J D Delhanty, Jadey D Raben, Yoëlle Goos, Anke McLuskey-Dankbar, Youri Boon, Rene de Vries, Jan von der Thüsen, A H Jan Danser, Anton J M Roks, Jenny A Visser

**Affiliations:** Division of Vascular Medicine and Pharmacology, Department of Internal Medicine, Erasmus MC, Erasmus Medical Centre Rotterdam, 3000 CA Rotterdam, The Netherlands; Division of Endocrinology, Department of Internal Medicine, Erasmus MC, Erasmus Medical Centre Rotterdam, 3000 CA Rotterdam, The Netherlands; Division of Endocrinology, Department of Internal Medicine, Erasmus MC, Erasmus Medical Centre Rotterdam, 3000 CA Rotterdam, The Netherlands; Division of Vascular Medicine and Pharmacology, Department of Internal Medicine, Erasmus MC, Erasmus Medical Centre Rotterdam, 3000 CA Rotterdam, The Netherlands; Division Nephrology and Transplantation, Department of Internal Medicine, Erasmus MC, Erasmus Medical Centre Rotterdam, 3000 CA Rotterdam, The Netherlands; Division of Endocrinology, Department of Internal Medicine, Erasmus MC, Erasmus Medical Centre Rotterdam, 3000 CA Rotterdam, The Netherlands; Division of Vascular Medicine and Pharmacology, Department of Internal Medicine, Erasmus MC, Erasmus Medical Centre Rotterdam, 3000 CA Rotterdam, The Netherlands; Division of Vascular Medicine and Pharmacology, Department of Internal Medicine, Erasmus MC, Erasmus Medical Centre Rotterdam, 3000 CA Rotterdam, The Netherlands; Department of Pathology and Clinical Bioinformatics, Erasmus MC, Erasmus Medical Centre Rotterdam, 3000 CA Rotterdam, The Netherlands; Division of Vascular Medicine and Pharmacology, Department of Internal Medicine, Erasmus MC, Erasmus Medical Centre Rotterdam, 3000 CA Rotterdam, The Netherlands; Division of Vascular Medicine and Pharmacology, Department of Internal Medicine, Erasmus MC, Erasmus Medical Centre Rotterdam, 3000 CA Rotterdam, The Netherlands; Division of Endocrinology, Department of Internal Medicine, Erasmus MC, Erasmus Medical Centre Rotterdam, 3000 CA Rotterdam, The Netherlands

**Keywords:** metabolic aging, sex-dimorphism, adipocytes, adipose tissue depots, transgelin

## Abstract

Metabolic aging is marked by changes in insulin sensitivity and adipose tissue remodeling, including the vasculature. Transgelin 1 (Tagln1), a marker for vascular smooth muscle cells (VSMC), is used as a promoter for *Cre-*driven gene knockout (KO) in mice. However, Tagln1 is also expressed in white adipocytes. We previously showed that *Tagln1-Cre*–driven KO of DNA repair endonuclease Ercc1 in mice (*Tagln1-Cre Ercc1* mice) leads to accelerated aging of VSMC and perivascular adipocytes, resulting in vascular dysfunction and lipodystrophic-like perivascular adipose tissue. The aim of this study was to investigate the consequences of Tagln1-driven Ercc1 deficiency on whole-body metabolism and adipose tissue morphology and function. *Tagln1-Cre Ercc1* mice did not show the usual age-related gain in fat mass. Adipose tissue depots from male *Tagln1-Cre Ercc1* mice shared similarities with adipose tissue depots from advanced-aged mice, including smaller white adipocyte size, decreased vascularization, increased oxidative stress, macrophage infiltration, and fibrosis compared with male wild-type (WT) mice. Female *Tagln1-Cre Ercc1* mice showed an upregulation of antioxidant genes, which might be a protective response resulting in minor changes in their adipose tissue depots compared with female WT mice. Male *Tagln1-Cre Ercc1* mice showed partially preserved adipose tissue function and improved insulin sensitivity. Interestingly, analyses showed that Tagln1 expression varied by sex and adipose tissue depot in WT mice. To conclude, we showed that accelerated aging induced by Ercc1 deficiency in Tagln1-dependent cell populations leads to white adipose tissue and brown adipose tissue remodeling and inflammation, as also observed in normal aging

Aging is associated with chronic metabolic disorders, including obesity and type 2 diabetes (1). During aging, adipose tissue undergoes significant changes, including redistribution and structural and functional remodeling, leading to adipose tissue dysfunction, which promotes chronic inflammation, altered adipokine secretion, and insulin resistance (1, 2). These age-related changes can be referred to as metabolic aging.

Healthy adipose tissue is responsible for maintaining systemic energy, glucose and lipid homeostasis (1). It consists of adipocytes, smooth muscle cells (SMC), endothelial cells (EC), neuronal and immune cells (3). Adipose tissue can be classified into brown adipose tissue (BAT), which is critical for energy expenditure (EE) and nonshivering thermogenesis (3-5), white adipose tissue (WAT), which is responsible for energy homeostasis and lipid storage, and an intermediate beige adipose tissue subtype (5). WAT is further subdivided by anatomical location into gonadal white adipose tissue (gWAT), inguinal white adipose tissue (iWAT), axillary white adipose tissue (aWAT), and visceral white WAT (6). The function of WAT and BAT is tightly regulated by insulin, lipids, hormones and adipokines, and the transport and distribution thereof rely on correct vascular perfusion of the adipose tissue depots (1, 7). Vascular aging could be a precipitating factor. Female WAT from young mice is more vascularized than male WAT (8), but it is unclear whether this sex difference persists with aging (8).

Age-related vascular dysfunction could contribute to impaired adipose tissue function and alterations in insulin sensitivity. The decline in insulin sensitivity and WAT function develops sex-specifically in middle-aged mice (9, 10). It is unclear what drives metabolic aging associated with adipose tissue changes, particularly the sequence of events, the involved adipocyte development factors, and the causal mechanism of aging require further research. Most studies on advanced aged mice exclude female mice (9-12), leaving it unclear whether sex differences persist until very old age. Mouse models in which aging is driven by a specific causal mechanism and controlled by an adipocyte development factor could be instrumental for untangling age-related mechanistic changes in the adipose tissue depots.

One driver of metabolic aging is genomic instability caused by accumulated DNA damage (13), triggering a DNA damage response, which features increased cellular stress, senescence, and inflammation [reviewed extensively elsewhere (14)]. KO of DNA repair endonuclease *Ercc1* induces accelerated DNA damage accumulation and thereby premature aging in mice (15). Moreover, Ercc1 KO induces the accumulation of senescent cells, which are marked by increased expression of cyclin-dependent kinase inhibitor 1A (p21), plasminogen activator inhibitor (Pai-1), and pro-inflammatory markers monocyte chemoattractant protein 1 (Mcp-1), Il-6, and Tnf-α (16-18). The upregulation of these inflammatory markers is also characteristic of chronic low-grade inflammation during aging (19, 20). Furthermore, it has also been shown that Ercc1 KO in mice activates antioxidant genes that are involved, among others, in glutathione and quinone metabolism (21, 22). In cell-specific *Ercc1* KO mice driven by transgelin 1 (Tagln1)–Cre (*Tagln1-Cre Ercc1*), we previously showed vascular dysfunction and low perivascular adipose tissue (PVAT) mass at the age of 22 weeks (16, 23). Tagln1, also known as smooth muscle 22 alpha, is an established SMC marker and is frequently used as a promoter to drive SMC-specific Cre (24, 25). Tagln1 supports the structural integrity and stability of the cytoskeleton by crosslinking actin (25). However, Tagln1 was recently reported to be a novel marker for white adipocyte precursors (26), where it plays a role during adipocyte differentiation (26). Moreover, lower PVAT mass has been reported for two independent *Tagln1-Cre*–driven KO mouse models (23, 27). Thus, *Tagln1-Cre Ercc1* mice could reveal important aging-related pathophysiological mechanisms in adipose tissues in which Tagln1-controlled cell differentiation plays a role, and their impact on whole-body metabolic changes.

Here, we investigate the impact of Ercc1 deficiency driven by *Tagln1-Cre* on EE, specifically adipose tissue function and morphology other than PVAT. Given the lack of data on female metabolic aging, we also assessed potential sex differences in *Tagln1-Cre Ercc1* mice.

## Methods

### Mouse model

Mice were bred as described previously (16). In brief, the Cre-loxP system was used to generate *Tagln1-Cre*–driven *Ercc1* KO mice (*Tagln1-Cre Ercc1*) by cross-breeding of *Tagln1*-promoter (Tagln^tm2(Cre)Yec^, 118 c57Bl/6 background, The Jackson Laboratory) with Ercc1^flox/−^ (Ercc1^tm2Dwm^, FVB 119 background, Erasmus MC colony) mice. The corresponding Cre^+/−^::Ercc1^flox/+^ healthy wild-type littermates (WT) were used as control WT mice. Only Cre-positive mice were used for the WT control group. All mice had access to water and food (complete universal vegetal diet, DS801727G10R, SDS diets) ad libitum. At the age of 8 weeks, 7 male WT, 6 male *Tagln1-Cre Ercc1*, 6 female WT, and 7 female *Tagln1-Cre Ercc1* mice were analyzed. At the age of 22 weeks, 23 male WT, 19 male *Tagln1-Cre Ercc1*, 8 female WT, and 10 female *Tagln1-Cre Ercc1* mice were divided over several experiments (see *in vivo* measurements discussed later). Mice were euthanized by exsanguination while deeply anesthetized under 3% isoflurane at the age of 8 or 22 weeks. BAT and WAT depots (gWAT, aWAT, and iWAT) were isolated, weighed, with one part stored in paraformaldehyde and the other part snap-frozen for mRNA analysis as described previously (28, 29). The iWAT depot refers to the posterior subcutaneous WAT, and the aWAT depot refers to the anterior subcutaneous WAT.

Animal experiments were performed at the Erasmus Laboratory Animal Science Center Rotterdam following the guidelines from Directive 2010/63/EU. All studies were approved by the National Animal Care Committee and the Erasmus University Medical Center Rotterdam.

### Body composition and intraperitoneal glucose tolerance test

At the age of 8, 12, 16, and 20 weeks, 11 male WT, 9 male *Tagln1-Cre Ercc1*, 9 female WT, and 10 female *Tagln1-Cre Ercc1* mice were weighed, and their body composition was assessed by EchoMRI™ (100 V Whole Body Composition Analyzer, EchoMRI LLC) in the morning. Afterwards, mice were fasted (no food, with water ad libitum) for 5 hours, followed by an intraperitoneal glucose tolerance test (IPGTT) as described previously (28). In brief, fasting glucose was measured with a glucometer (FreeStyle Freedom Lite glucometer, Abbott) followed by intraperitoneal injection of a glucose bolus (2 g/kg body weight). Blood glucose was measured 15, 30, 60, and 120 minutes after injection. The average plasma glucose levels were then plotted per group over time. To assess the area under the curve, glucose levels were normalized to fasting glucose level per mouse, plotted over time, and the area under the curve was calculated with GraphPad Prism (Version 8.0.1).

### Hormone measurements

Fasting insulin was measured in blood plasma collected at baseline of the IPGTT with the Ultra-Sensitive Rat insulin ELISA kit (Crystal Chem cat no. 62300, RRID:AB_3662575) following the manufacturer's instructions. From fasting glucose and insulin levels, the quantitative insulin sensitivity check index (QUICKI) was calculated using the formula QUICKI = 1/[(log(fasting insulin) + log(fasting glucose)] as described previously (30).

Moreover, leptin, IL-6 and TNF-α plasma levels were measured in blood collected in EDTA-coated tubes during the sacrifice of the mice. After centrifugation for 5 minutes at 2500 rpm and 4 °C, the EDTA plasma was stored at −20 °C for measurement. Leptin levels were quantified with the Leptin Mouse ProcartaPlex™ Simplex Kit (Thermo Fisher Scientific cat no. EPX01A-26036-901, RRID:AB_2575954) and IL-6 and TNF-α with the High Sensitivity 5-Plex Mouse ProcartaPlex™ Panel (Thermo Fisher Scientific cat no. EPXS050-22199-901, RRID:AB_2576189) on a Bio-Plex MAGPIX multiplex reader (Bio-Rad, Laboratory Medical Immunology, Erasmus MC) according to the manufacturer's instructions.

### Indirect calorimetry assessment

One week before sacrifice (21 weeks of age), the mice (11 male WT, 7 male *Tagln1-Cre Ercc1*, 9 female WT, and 9 female *Tagln1-Cre Ercc1*) were housed individually in a metabolic cage system (Promethion, Sable Systems International). The cages (Greenline, Tecniplast) contained woodchip bedding (Lignocel BK 8/15, J. Rettenmaier & Söhne GmbH) and were fitted with a water bottle, food hopper, and a nest. These were designed to measure water, food intake, and body weight, respectively, via load cells located in the lids of the cages. The horizontal and vertical locations of the mice in each cage were detected by an infrared beam grid, and data were converted to locomotor activity. All mass and positional measurements were detected at 1 Hz. Cage air influx was set at 2 L/min, and volumes of consumed oxygen (V̇O_2_), produced carbon dioxide (V̇CO_2_), and water vapor (V̇H2O) were measured at 5-minute intervals, from which EE and respiratory exchange ratio (RER) were derived. Data from the system were collected using MetaScreen (Version 2.3; Sable Systems International) software and processed with MacroInterpreter (version 23.9; Sable Systems International). Indirect calorimetry data (1-hour bins) from a 48-hour period following approximately 24 hours of habituation were analyzed with CalR (version 1.3) (31). Two-way analysis of covariance (IBM SPSS Version 28) with lean mass as a covariate was used to analyze effects of sex and genotype on EE. Two-way ANOVA (GraphPad Prism 10.0.1) was used to evaluate differences in RER and locomotor activity between the 4 sex/genotype experimental groups. *P* < .05 was considered statistically significant.

### Acute insulin test

For the acute insulin test, only male mice were analyzed, because no differences were observed between female WT and *Tagln1-Cre Ercc1* mice in the IPGTT. Mice were fasted for 5 hours and weighed, and the appropriate amount of insulin/body weight was calculated. Male mice were injected with 1% (w/v) BSA in saline (vehicle) or 1 IU/kg insulin (WT mice, n = 6 per group; *Tagln1-Cre Ercc1* mice, n = 5 per group). Mice were sacrificed 15 minutes after injection, and tissues were dissected and snap frozen in liquid nitrogen for later analysis.

### Protein expression

Total and phosphorylated AKT protein expression was evaluated by Western blot. Proteins were extracted from insulin-stimulated or vehicle gWAT, aWAT, iWAT, and BAT in homogenization buffer (0.3 mol/L sucrose, 50 mmol/L Tris-HCl pH 7.5, 1 mmol/L EDTA, 1 mmol/L EGTA, 1 mmol/L sodium orthovanadate, 50 mmol/L sodium fluoride, 1 mmol/L DTT, 1 mmol/L phenylmethylsulfonyl fluoride, 1% [v/v] Triton X-100, phosphatase inhibitor cocktail 3 [Sigma-Aldrich] and cOmplete^TM^ Protease Inhibitor Cocktail [Roche]). Samples were normalized to the lowest protein concentration with 1 × Laemmli buffer and incubated for 10 minutes at 65 °C. A total of 20 µg (BAT and gWAT) or 25 µg (aWAT and iWAT) protein per sample was loaded on a 4% to 20% Criterion™ TGX™ Precast Midi Protein Gel (Bio-Rad) together with a protein ladder (PageRuler™ Plus Prestained Protein Ladder, Thermo Fisher Scientific) and transferred to a polyvinylidene difluoride membrane (Trans-Blot Turbo 0.2 µm PVDF Transfer Pack, Bio-Rad) with the Trans-Blot Turbo Transfer System (Bio-Rad). Membranes were blocked with 5% (w/v) BSA or nonfat milk in Tris-buffered saline containing 1% [v/v] Tween 20 (TBS-T) buffer for 2 hours at room temperature (RT) according to the primary antibody, followed by overnight incubation in the primary antibodies rabbit anti-total AKT (1:1000, Cell Signaling Technology cat no. 4691, RRID:AB_915783) and rabbit anti-phosphorylated AKT (Ser473) (1:2000, Cell Signaling Technology cat no. 4060, RRID:AB_2315049) or rabbit anti-glyceraldehyde-3-phosphate dehydrogenase (GAPDH; 1:10 000; Proteintech cat no. 10494-1-AP, RRID:AB_2263076) as loading control (diluted in 5% [w/v] BSA or nonfat milk [GAPDH] in TBS-T buffer) at 4 °C. The membranes were then incubated for 1 hour at RT in the secondary antibody (goat anti-rabbit-horseradish peroxidase [HRP] conjugate [Bio-Rad, 1:3000; Bio-Rad cat no. 170-6515, RRID:AB_11125142] diluted in 5% [w/v] nonfat milk in TBS-T buffer) and visualized with the Clarity Western ECL substrate (Bio-Rad) following the manufacturer's instructions using an Amersham Al600. Membranes were exposed for a timeframe so that all bands were clearly visible but not overexposed. Moreover, colorimetric pictures of the protein ladder per membrane were taken. Pictures were saved in TIFF format, and the signals of the bands for the proteins were quantified in Image Studio Lite (version 5.2.5). Data are presented as the ratio of protein of interest/GAPDH for individual proteins or as the ratio of phosphorylated/total AKT as average per group ± SEM.

### mRNA expression

Samples from gWAT, aWAT, iWAT, BAT, liver, and skeletal muscle were homogenized in TRIzol (Thermo Fisher), and RNA was isolated according to the manufacturer's instructions. cDNA was synthesized according to the manufacturer's protocol (Maxima H Minus First Strand cDNA Synthesis Kit, Thermo Scientific). Quantitative PCR was performed with the SYBR Green PCR Master Mix (ThermoFisher) measured as duplicates per sample (1 × PCR Master Mix, 200 nmol/L forward primer, 200 nmol/L reverse primer, 10 ng cDNA). *β-Actin*, *Gapdh*, and *Rpl13a* were used as reference genes measured in the CFX Opus Real-Time PCR system (Bio-Rad). Data were analyzed with the 2^ΔΔCt^ method, expressed as relative quantity. Used primers (self-designed and ordered from IDT) are listed in Table 1.

**Table 1:** Primers used for quantitative PCR

Gene	Primer sequence
*Adipoq* forward	GCACTGGCAAGTTCTACTGCAA
*Adipoq* reverse	GTAGGTGAAGAGAACGGCCTTGT
*Atgl* forward	ACCATCTGCCTTCCAGACTGT
*Atgl* reverse	TAGGGCCTCACTGTAGGTGG
*β-Actin* forward	CACTGTCGAGTCGCGTCC
*β-Actin* reverse	TCATCCATGGCGAACTGGTG
*Bax* forward	CAAACTGGTGCTCAAGGCCC
*Bax* reverse	TCTTGGATCCAGACAAGCAGC
*C/ebpa* forward	CTCTTCACTAACGGCTGGGG
*C/ebpa* reverse	TGGACAACTAGGGGAGAGGG
*C/edpd* forward	GCTGAGCGACGAGTACAAGAT
*C/edpd* reverse	TGCTGCGTCTCCAGGTTG
*Cidea* forward	AAGGCCGTGTTAAGGAATCTG
*Cidea* reverse	CTGCTCTTCTGTATCGCCCA
*Dlk1* forward	AAGGCCAGTACGAATGCTCC
*Dlk1* reverse	TGGCAGGGAGAACCATTGAT
*Dio2* forward	CCTAGATGCCTACAAACAGGTTA
*Dio2* reverse	CCCATCAGCGGTCTTCTCC
*eNOS* forward	GCAGGTATTTGATGCTCGGG
*eNOS* reverse	TGGCTGAACGAAGATTGCCT
*Ercc1 forward*	CATTTGGATCCCTGGAACAGC
*Ercc1 reverse*	TAGTACTTCAAAGAGCCTGCGG
*Fabp4* forward	GGGAACCTGGAAGCTTGTCT
*Fabp4* reverse	GGCAAAGCCCACTCCCAC
*Fasn* forward	GAGCCCAGACAGAGAAGAGC
*Fasn* reverse	TGTCCACACCACCAATGAGG
*Fkbp5* forward	TGTTCAAGAAGTTCGCAGAGC
*Fkbp5* reverse	CCTTCTTGCTCCCAGCTTT
*Gapdh* forward	GTGCAGTGCCAGCCTCGT
*Gapdh* reverse	GAAGGGGTCGTTGATGGCAA
*Gsta1* forward	CTTCTGACCCCTTTCCCTCT
*Gsta1* reverse	ATCCATGGGAGGCTTTCTCT
*Gstt2* forward	CGAGCAATTCTCCCAGGTGA
*Gstt2* reverse	TATTCGTGGACTTGGGCACG
*Hmox1* forward	CAGGTGATGCTGACAGAGGA
*Hmox1* reverse	ATGGCATAAATTCCCACTGC
*Hsl* forward	GACCATCAACCGACCAGGAG
*Hsl* reverse	TGGCAGGTGTGAACTGGAAG
*Il6* forward	TCCAGTTGCCTTCTTGGGAC
*Il6* reverse	GTGTAATTAAGCCTCCGACTTG
*Irs-1* forward	ACTGGACATCACAGCAGAATG
*Irs-1* reverse	AAGACGTGAGGTCCTGGTTG
*Lep* forward	ACCCCATTCTGAGTTTGTCC
*Lep* reverse	TCCAGGTCATTGGCTATCTG
*Mcp-1* forward	AGCTGTAGTTTTTGTCACCAAGC
*Mcp-1* reverse	TGTCTGGACCCATTCCTTCTTG
*Nfe2l2* forward	AGGACATGGAGCAAGTTTGG
*Nfe2l2* reverse	TCTGTCAGTGTGGCTTCTGG
*Nqo1* forward	TTCTGTGGCTTCCAGGTCTT
*Nqo1* reverse	AGGCTGCTTGGAGCAAAATA
*P21* forward	CAGACCAGCCTGACAGATTTC
*P21* reverse	GTTTTCTCTTGCAGAAGACCAATCT
*P53* forward	CTCCGAAGACTGGATGACTGC
*P53* reverse	TCCATGCAGTGAGGTGATGG
*Pai-1* forward	GCACAACCCGACAGAGACAA
*Pai-1* reverse	ATGAAGGCGTCTCTTCCCAC
*Pgc1α* forward	CAGGAACAGCAGCAGAGACA
*Pgc1α* reverse	CCCTTGGGGTCATTTGGTGA
*Pparγ* forward	GGGATGTCTCACAATGCCATC
*Pparγ* reverse	ATCTCCGCCAACAGCTTCTC
*Prdm16* forward	GGGAAGTCACAGGAGGACAC
*Prdm16* reverse	TCGCTCCTCAACACACCTCC
*Puma* forward	TACGAGCGGCGGAGACAAG
*Puma* reverse	GTGTAGGCACCTAGTTGGGC
*Rpl13a* forward	ACAGCCACTCTGGAGGAGAA
*Rpl13a* reverse	GCCTGTTTCCGTAACCTCAAG
*Scd1* forward	ACCTGCCTCTTCGGGATTTT
*Scd1* reverse	CAGCCGTGCCTTGTAAGTTC
*Srebf1* forward	CAGCAGGTCCCAGTTGTACT
*Srebf1* reverse	GGAGCCAGGGTGCTGATG
*Sirt-1* forward	AGCGGCTTGAGGGTAATCAA
*Sirt-1* reverse	GAGTATACCTCAGCACCGTGG
*Slc2a4* forward	TATTGCAGCGCCTGAGTCTT
*Slc2a4* reverse	GGTTCCCCATCGTCAGAGC
*Tagln1* forward	GGCTGTGACCAAAAACGATGG
*Tagln1* reverse	ATGCTCCTGGGCTTTCTTCATAA
*Tnfα* forward	GTCCCCAAAGGGATGAGAAGTT
*Tnfα* reverse	GCTACAGGCTTGTCACTCGAA
*Ucp1* forward	GGCCTCTACGACTCAGTCCA
*Ucp1* reverse	TAAGCCGGCTGAGATCTTGT
*Ve-cadherin* forward	CATCAAACCCACGAAGTCCC
*Ve-cadherin* reverse	TTTTGCCTGAAGTGCTGCTC
*Vegf-α* forward	AAAACACAGACTCGCGTTGC
*Vegf-α* reverse	CCTTGGCTTGTCACATCTGC

Adipoq, adiponectin; Atgl, Adipose triglyceride lipase; β-Actin, beta-actin; Bax, BCL2-associated X protein; C/ebpa, CCAAT/enhancer-binding protein alpha; C/edpd, CCAAT/enhancer-binding protein delta; Cidea, Cell Death Inducing DFFA Like Effector A; Dlk1, Delta Like Non-Canonical Notch Ligand 1; Dio2, Iodothyronine Deiodinase 2; eNOS, endothelial nitric oxide synthase; Ercc1, Excision Repair Cross-Complementation Group; Fabp4, Fatty Acid Binding Protein 4; Fasn, Fatty Acid Synthase; Fkbp5, FK506 Binding Protein 5; Gapdh, Glyceraldehyde-3-Phosphate Dehydrogenase; Gsta1, Glutathione S-Transferase Alpha 1; Gstt2, Glutathione S-Transferase Theta 2; Hmox1, Heme Oxygenase 1; Hsl, Lipase E, Hormone Sensitive Type; Il6, Interleukin 6; Irs-1, Insulin receptor substrate 1; Lep, Leptin; Mcp-1, C-C Motif Chemokine Ligand 2; Nfe2l2, Nuclear Factor (Erythroid-Derived 2)-Like 2; Nqo1, NAD(P)H Quinone Dehydrogenase 1; P21 (Cdkn1a), Cyclin Dependent Kinase Inhibitor 1A; P53, Tumor Protein P53; Pai-1, Serpine 1; Pgc1α, Peroxisome Proliferator-Activated Receptor Gamma Coactivator 1 Alpha; Pparγ, Peroxisome Proliferator-Activated Receptor Gamma; Prdm16, PR/SET Domain 16; Puma, BCL2 Binding Component 3; Rpl13a, Ribosomal Protein L13a; Scd1, Stearoyl-CoA Desaturase; Srebf1, Sterol Regulatory Element Binding Transcription Factor 1; Sirt-1, Sirtuin 1; Slc2a4, Solute Carrier Family 2 Member 4; Tagln1, Transgelin 1; Tnfα, Tumor Necrosis Factor α; Ucp1, Uncoupling protein 1; Ve-cadherin (Cdh5), Vascular endothelial-cadherin; Vegf-α, Vascular endothelial growth factor A.

### Histology

Paraffin-embedded adipose tissue and liver samples, fixed in paraformaldehyde, were sectioned into 5-µm-thick sections using a microtome. These sections were mounted 1:20 on glass slides and stained with hematoxylin and eosin (H&E). The adipose tissue sections from the 22-week-old mice were also stained with Picrosirius Red to assess the collagen content. Representative pictures per group were included in the manuscript. Whole slide scans were performed with a digital imaging system (Hamamatsu NanoZoomer 2.0HT Digital Slide Scanner).

### Adipocyte size measurement and lipid area quantification

Representative images from 3 H&E-stained sections per depot and animal were randomly selected and captured using viewing software (NDP.view2). For BAT and aWAT, lipid area quantification was performed in an automated manner in ImageJ (Fiji, Version 2.16.0) using a macro that contained image conversion to black and white (8-bit) and a threshold of 210 to 255 for BAT and 220 to 255 for aWAT. Area (µm) and area fraction (%) were the outcome measurements, which were exported to Excel. Lipid area was calculated as the percentage of total area and presented as average ± SEM per adipose tissue depot, sex, and genotype.

For gWAT and iWAT, adipocyte size was determined in the H&E-stained sections in ImageJ (Fiji, Version 2.16.0) as described previously (32). In brief, the images were first converted to black and white, and a threshold was set manually per adipose tissue depot according to the best differentiation between adipocytes. The following features were used for the automated size determination: Circularity was set at 0.2 to 1.00, and size was set at 100 to 50,000 µm. Data are presented as average ± SEM per adipose tissue depot, sex, and genotype.

### Immunohistochemistry

Immunohistochemical staining for F4/80 and cleaved Caspase-3 was performed in gWAT sections from 2 male and 2 female mice per group. For liver tissue, the staining for TAGLN1 and CD31 was performed on sections from 2 male and 2 female 22-week-old mice. Sections (5 µm thick) were mounted, deparaffinized, blocked for endogenous peroxidase and alkaline phosphatase activity, and washed with demi water. Following antigen retrieval in 1 × Tris-EDTA buffer (pH 9.0), sections were cooled down, rinsed in 1 × PBS and blocked for nonspecific binding with 5% (v/v) normal goat serum (X090710-8, Agilent Technologies) in 1 × PBS for 30 minutes. Then, sections were incubated with primary antibodies rabbit anti-F4/80 (1:400; Cell Signaling, 70076; RRID:AB_2799771), rabbit anti-cleaved Caspase-3 (1:100; Cell Signaling, #9661; RRID:AB_2341188), rabbit anti-Tagln1 (1:400; Abcam, cat no. ab14106, RRID:AB_443021) or rabbit anti-CD31 (1:2000; Abcam, cat no. ab182981). Sections were incubated at 4 °C overnight and washed with 1 × PBS. Next, sections were incubated for 30 minutes at RT with BrightVision antirabbit HRP-conjugated (ready-to-use, PVR55HRP, VWR) as the secondary antibody, washed with 1 × PBS, and the antibody binding sites were revealed with 3,3-diaminobenzidine tetrahydrochloride. Finally, all sections were counterstained with hematoxylin.

TAGLN1 staining on gWAT, aWAT, iWAT, and BAT sections from 2 male and 2 female WT mice was performed according to the protocol as previously described, but with the following adjustments: antigen retrieval was performed in 1 × citrate buffer (pH 6.0), sections were blocked for nonspecific binding with 10% (v/v) normal goat serum (S-1000-20, VectorLabs) in 5% (w/v) BSA, and blocked for endogenous biotin. Sections were incubated with primary antibody rabbit anti-Tagln1 (1:200, Abcam cat no. ab14106, RRID:AB_443021) and then incubated with biotinylated antirabbit (1:200, Vector Laboratories cat no. BA-1000, RRID:AB_2313606) as secondary antibody. Afterward, the sections were incubated with streptavidin-biotin-peroxidase complex (avidin-biotin complex-HRP, 1:200 dilution, Vector Laboratories cat no. PK-6100, RRID:AB_2336819), the antibody binding sites were revealed with 3,3-diaminobenzidine tetrahydrochloride, and all sections were counterstained with hematoxylin.

### Liver lipid analysis

The liver H&E-stained sections were blindly scored regarding compactness of the tissue and the relative presence of lipids with the following criteria per category: 1, compact, few lipids; 2, compact, some lipids; 3, fairly compact, some lipids (mix); 4, less compact, some lipids; 5, less compact, more lipids; and 6, not compact, many lipids.

### Triglyceride assay

The liver triglyceride content was analyzed with the Triglyceride Colorimetric Assay Kit (Elabscience; #E-BC-K238-96) in homogenized tissue. Twenty milligrams of liver was washed in cold PBS buffer and then homogenized on ice in 180 µL PBS buffer with added protease inhibitor (cOmplete, EDTA-free Protease Inhibitor cocktail tablets, REF 11873580001, Roche). After centrifugation (10 minutes, 4 °C, 12 000 × g), the supernatant was collected and immediately used for the measurements at 510 nm with the CLARIOstar plate reader (BMG LABTECH; RRID:SCR_026330) following the kit manual. The resulting absorption values were then normalized to protein content in the individual samples, measured in a Bradford assay (CLARIOstar plate reader, BMG LABTECH).

### Statistics

Results are presented as average ± SEM for all groups except for mRNA expression, where results are presented as geometric mean ± SD of geometric mean (33). Results presented in column graphs were either evaluated by an unpaired Student *t* test (when 2 groups were compared) or 2-way ANOVA with a post hoc Bonferroni multiple comparisons test when assessing how sex and genotype (2 independent categorical variables) affect a dependent continuous variable (eg, leptin levels; GraphPad Prism 8.0.1). For each gene panel, the data were analyzed by 2-way ANOVA with a post hoc Bonferroni multiple comparisons test to correct for the number of genes analyzed. IPGTT data were analyzed with 2-way ANOVA with a post hoc Bonferroni multiple comparisons test and a general linear model with repeated measures (GraphPad Prism 8.0.1).

## Results

### Effects of *Tagln1-Cre Ercc1* KO on general metabolic parameters

Body weight and composition were recorded starting at the age of 8 weeks until the age of 20 weeks (Fig. 1A), since at these ages we previously observed vascular dysfunction in this mouse model (16, 23). We hypothesized that potential metabolic changes might occur around the same age. In contrast to WT mice, body weight of *Tagln1-Cre Ercc1* mice did not show the age-related increase as observed in the WT mice but remained unchanged over time in both sexes (Fig. 1A, left panel). Furthermore, *Tagln1-Cre Ercc1* mice were not able to gain fat mass over time, in contrast to their corresponding WT littermates (Fig. 1A, middle panel). The difference in fat mass was particularly pronounced in male mice starting already after 8 weeks, whereas in female mice the genotype effect became statistically significant after 12 weeks. However, there was no statistical difference between male and female *Tagln1-Cre Ercc1* mice regarding total fat mass. To more clearly analyze these changes, we compared the endpoints at the age of 8 and 20 weeks per sex. Male 20-week-old *Tagln1-Cre Ercc1* mice showed a trend of losing fat mass compared with male 8-week-old *Tagln1-Cre Ercc1* mice without reaching statistical significance (*P* = .0588), while male WT mice strongly gained fat mass over time (*P* < .0001). Female *Tagln1-Cre Ercc1* mice neither gained nor lost fat mass over time (*P* > .9999), which is in contrast to female WT mice, which showed an increase in fat mass over time (*P* < .0001). Male WT mice displayed a high body weight and fat mass (34), reminiscent of an obese-like phenotype (35-37), which has been reported previously for male mice from this strain (34). However, female WT mice did not show a similar high gain in body weight at 22 weeks of age, which is in line with a previous report (38). This sex difference in body weight likely contributed to the more pronounced genotype effect in male mice. Absolute lean mass increased over time in male and female WT mice. In contrast, in male and female *Tagln1-Cre Ercc1* mice, lean mass remained relatively constant over time (Fig. 1A, right panel), resulting in significantly lower absolute lean mass in *Tagln1-Cre Ercc1* mice compared with WT mice for both sexes at the age of 16 and 20 weeks.

**Figure 1:**
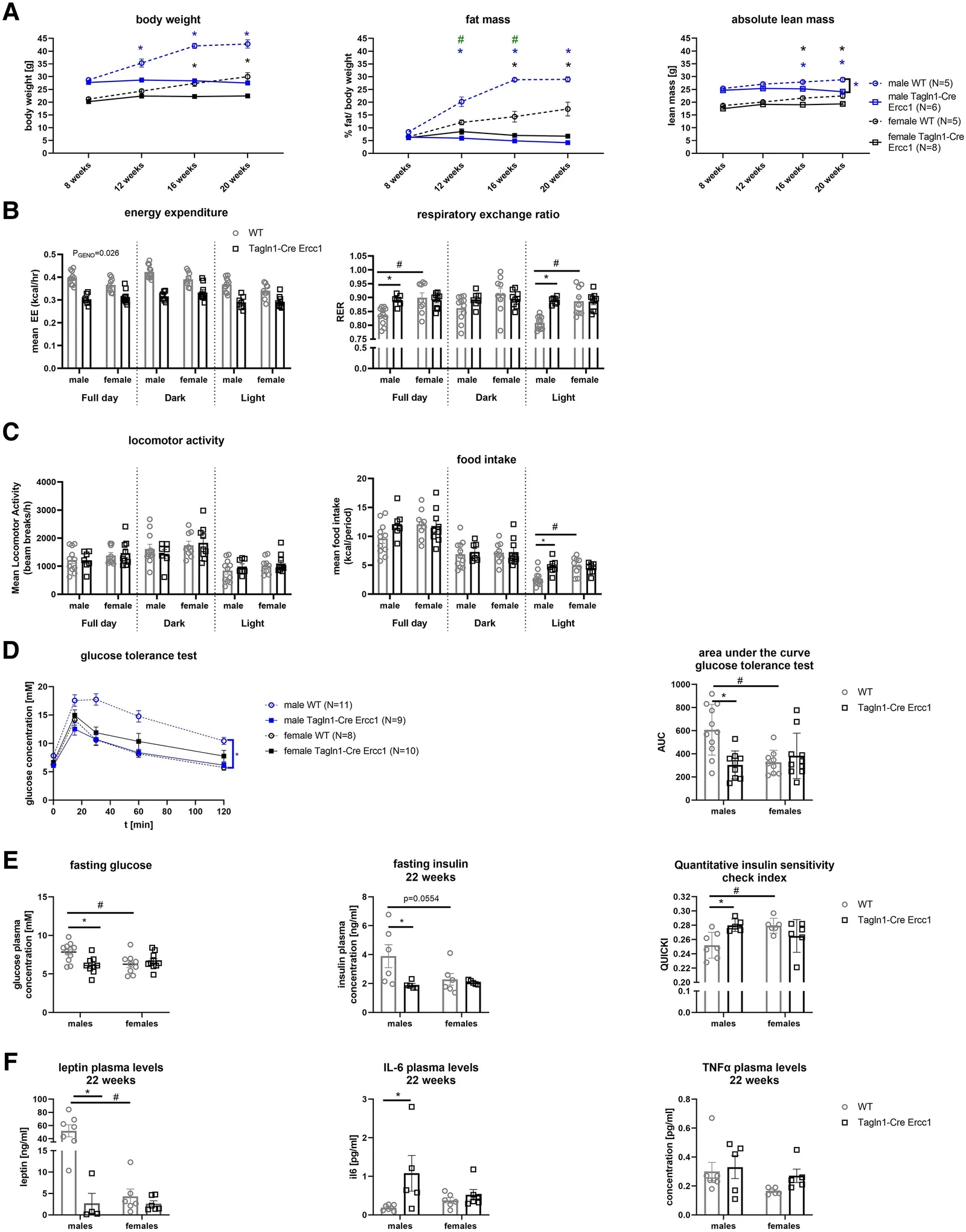
Metabolic parameters measured in wild-type (WT) and *Tagln1-Cre*–driven *Ercc1* knockout (KO; *Tagln1-Cre Ercc1*) mice. (A) Body composition measurements (body weight [left], fat mass [middle], and lean mass [right]) recorded over time in WT (11 males and 9 females) and *Tagln1-Cre Ercc1* mice (9 males and 10 females). There is a significant interaction of genotype × sex with *P* < .05 by 2-way ANOVA. ^#^Significant effect of sex (green hashtag for WT mice). *Significant effect of genotype (blue asterisk for male mice and black asterisk for female mice) by post hoc test. (B) Energy expenditure (EE) (left) and respiratory exchange ratio (RER) (right). (C) Locomotor activity (left) and food intake (right) assessed in 21-week-old WT (11 males and 9 females) and *Tagln1-Cre Ercc1* mice (7 males and 9 females) by indirect calorimetry experiments. Two-way analysis of covariance with lean mass as a covariate was used to analyze effects of sex and genotype on EE. Two-way ANOVA was used to evaluate differences in RER and locomotor activity between the 4 sex/genotype experimental groups. *P* < .05 was considered statistically significant. ^#^: significant effect of sex and *: significant effect of genotype. (D) Glucose tolerance test performed in 21-week-old WT (11 male and 9 female mice) and *Tagln1-Cre Ercc1* mice (9 male and 10 females) and *Tagln1-Cre Ercc1* mice. There is a significant interaction of genotype × sex with *P* < .05 by 2-way ANOVA. ^#^Significant effect of sex. *Significant effect of genotype by post hoc test. The left graph was additionally evaluated using a general linear model with repeated measures. (E) Fasting glucose and plasma insulin levels measured before the glucose tolerance test in 21-week-old WT and *Tagln1-Cre Ercc1* mice and quantitative insulin sensitivity check index (QUICKI) calculated from fasting glucose and insulin levels. There is a significant interaction of genotype × sex with *P* < .05 by 2-way ANOVA. ^#^Significant effect of sex. *Significant effect of genotype by post hoc test. (F) Leptin, IL-6, and TNF-α plasma levels measured in blood plasma from 22-week-old WT (7-8 males and 6 females) and *Tagln1-Cre Ercc1* mice (4-5 males and 6 females). There is a significant interaction of genotype × sex with *P* < .05 by 2-way ANOVA. ^#^Significant effect of sex. *Significant effect of genotype by post hoc test.

Indirect calorimetry was used to assess changes in EE, RER, mean locomotor activity, and food intake (Fig. 1B and 1C). EE (full day and dark phase) was significantly decreased in *Tagln1-Cre Ercc1* mice compared with WT mice in both sexes (Fig. 1B, left panel). Furthermore, we observed a significantly higher RER in male *Tagln1-Cre Ercc1* mice compared with male WT mice (full day and light phase) (Fig. 1B, right panel). There was neither a genotype nor a sex effect on locomotor activity (Fig. 1C, left panel). Food intake was significantly increased in male *Tagln1-Cre Ercc1* mice compared with male WT mice in the light phase, but there was no genotype effect during the dark phase or the whole assessment period (Fig. 1C, right panel).

To evaluate potential changes in glucose sensitivity, we performed an IPGTT. Male *Tagln1-Cre Ercc1* mice were significantly more glucose tolerant than male WT mice, while there were no differences between female *Tagln1-Cre Ercc1* and WT mice (Fig. 1D). Furthermore, compared with male WT mice, fasting glucose and plasma insulin levels were lower in male *Tagln1-Cre Ercc1* mice, suggesting they have greater insulin sensitivity (Fig. 1E, left and middle panels). This was supported by the QUICKI, which was higher in male *Tagln1-Cre Ercc1* mice than in male WT mice (Fig. 1E, right panel).

Changes in body composition and glucose metabolism also affected circulating levels of the adipokine leptin and the proinflammatory markers (Fig. 1F). Plasma leptin levels were significantly lower in male *Tagln1-Cre Ercc1* mice compared with male WT mice, but there was no difference between female *Tagln1-Cre Ercc1* and WT mice (Fig. 1F, left panel). We previously reported no genotype or sex effect in circulating adiponectin levels (23). Plasma IL-6 levels were significantly increased in male *Tagln1-Cre Ercc1* mice compared with male WT mice (Fig. 1F, middle panel), while no differences were observed between female *Tagln1-Cre Ercc1* and WT mice. Plasma levels of proinflammatory cytokine TNF-α were not different between the groups (Fig. 1F, right panel).

### Changes in WAT morphology in *Tagln1-Cre Ercc1* mice

Morphological changes in WAT depots from *Tagln1-Cre Ercc1* and WT mice were analyzed at the age of 8 weeks before onset of fat loss and at the age of 22 weeks when significantly decreased adipose tissue mass was observed independent of sex.

At the age of 8 weeks, the relative weights of all WAT depots were comparable between the groups (Fig. 2A), and there were only minor morphological changes in the WAT depots (Fig. 2B and 2C). The only difference was the denser iWAT from female *Tagln1-Cre Ercc1* mice compared with male *Tagln1-Cre Ercc1* mice (Fig. 2B, right panel), which was confirmed by a significantly decreased average adipocyte size (Fig. 2B and 2C, right panels).

**Figure 2:**
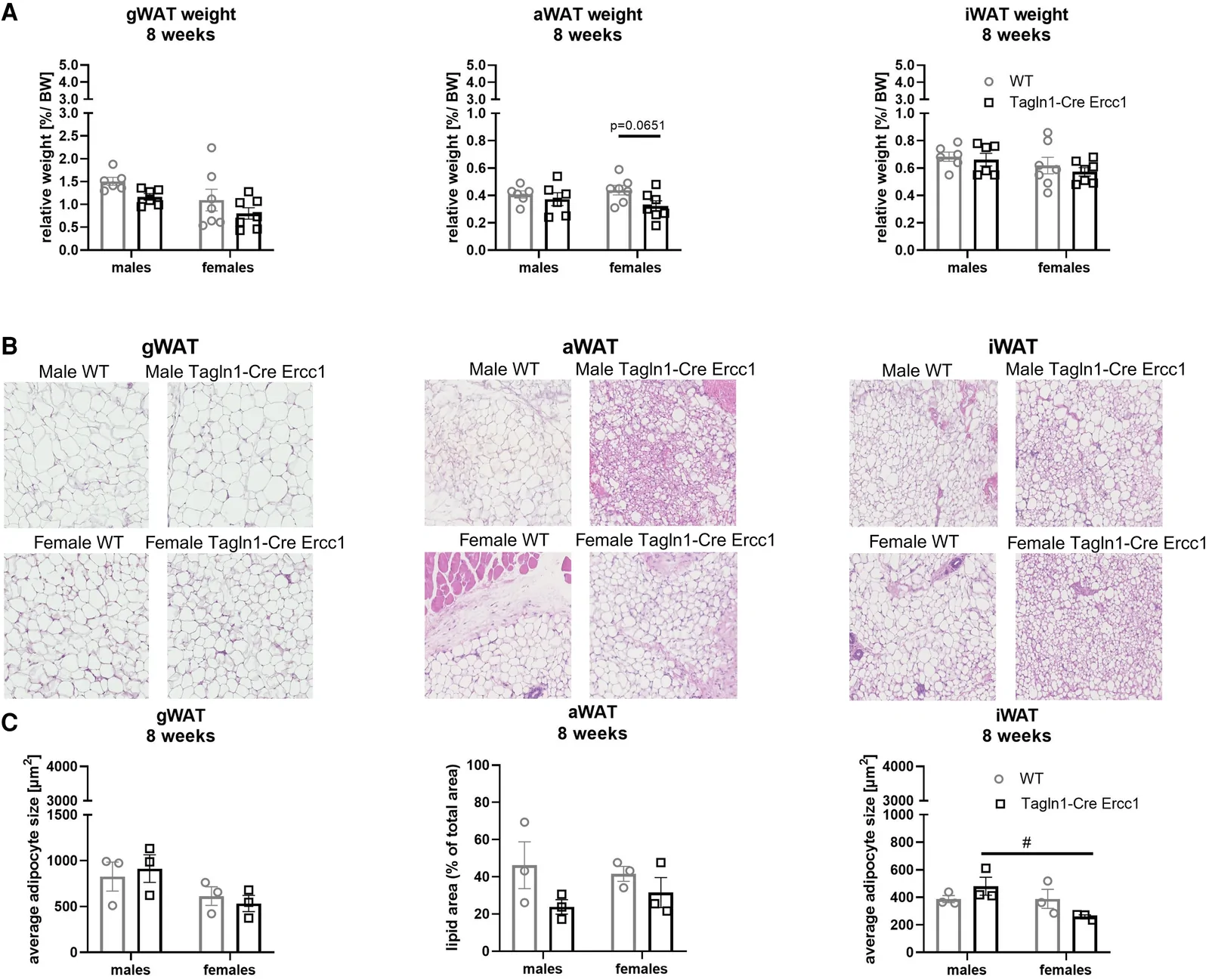
Morphological characterization of white adipose tissue (WAT) depots isolated from 8-week-old wild-type (WT) and *Tagln1-Cre*–driven *Ercc1* knockout (KO; *Tagln1-Cre Ercc1*) mice. (A) Relative WAT depot weights. (B) Hematoxylin and eosin (H&E) staining pictures to assess morphological changes in WAT. (C) Average adipocyte size (gWAT, iWAT) and lipid area (aWAT) calculated from H&E staining. There is a significant interaction of genotype × sex with *P* < .05 by 2-way ANOVA. ^#^Significant effect of sex. *Significant effect of genotype by post hoc test. Abbreviations: aWAT, axillary white adipose tissue; gWAT, gonadal white adipose tissue; iWAT, inguinal white adipose tissue.

In contrast, at the age of 22 weeks, the relative weights of all WAT depots from male *Tagln1-Cre Ercc1* mice were significantly decreased compared with male WT mice (Fig. 3A). Also, in female mice, the relative weights of gWAT (Fig. 3A, left panel) and iWAT (Fig. 3A, right panel) were significantly decreased in female *Tagln1-Cre Ercc1* mice compared with female WT mice, although the differences were less pronounced compared with male mice. All adipose tissue depots from male *Tagln1-Cre Ercc1* mice showed a much denser morphology compared with male WT mice (Fig. 3B). These observations were confirmed by calculating the average adipocyte size per depot, which was significantly decreased in gWAT of male *Tagln1-Cre Ercc1* mice compared with male WT mice (Fig. 3B and 3C, left panels). There was a similar trend in iWAT, but the differences failed to reach statistical significance (Fig. 3B and 3C, right panels). Given the dense appearance of aWAT at this age, it was not possible to accurately measure adipocyte size. Therefore, we calculated the lipid area as an estimate of average adipocyte size. This was significantly decreased in male *Tagln1-Cre Ercc1* mice compared with male WT mice (Fig. 3B and 3C, middle panels). In female *Tagln1-Cre Ercc1* mice, morphological changes in the adipose tissue depots were milder compared with male *Tagln1-Cre Ercc1* mice. Although we did observe a denser morphology in all WAT depots of female *Tagln1-Cre Ercc1* mice compared with female WT mice, only in the aWAT lipid area was it significantly decreased compared with female WT mice (Fig. 3B and 3C).

**Figure 3:**
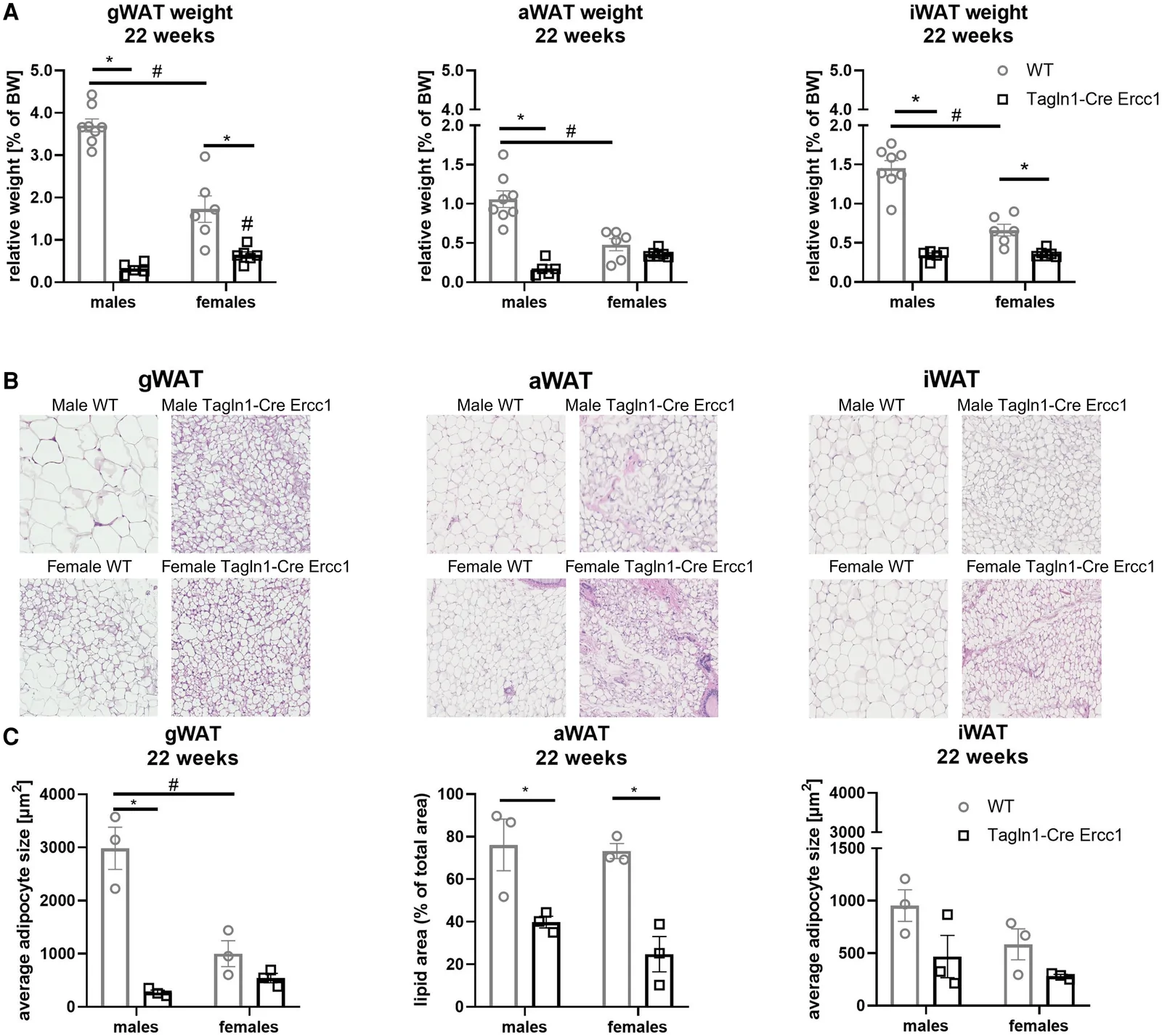
Morphological characterization of white adipose tissue (WAT) depots isolated from 22-week-old wild-type (WT) and *Tagln1-Cre*–driven *Ercc1* knockout (KO; *Tagln1-Cre Ercc1*) mice. (A) Relative WAT depot weights. (B) Hematoxylin and eosin (H&E) staining pictures to assess morphological changes in WAT. (C) Average adipocyte size (gWAT, iWAT) and lipid area (aWAT) calculated from H&E staining. There is a significant interaction of genotype × sex with *P* < .05 by 2-way ANOVA. ^#^Significant effect of sex. *Significant effect of genotype by post hoc test. Abbreviations: aWAT, axillary white adipose tissue; gWAT, gonadal white adipose tissue; iWAT, inguinal white adipose tissue.

To assess whether age-related fibrosis plays a role in the phenotype of *Tagln1-Cre Ercc1* mice, we performed Picrosirius Red staining in gWAT and iWAT to assess the collagen content (Fig. 4). We observed increased collagen deposition in the adipose tissue depots and around the arteries of gWAT and iWAT from *Tagln1-Cre Ercc1* mice compared with the WT littermates, which was most pronounced in iWAT from male *Tagln1-Cre Ercc1* mice compared with the other groups.

**Figure 4:**
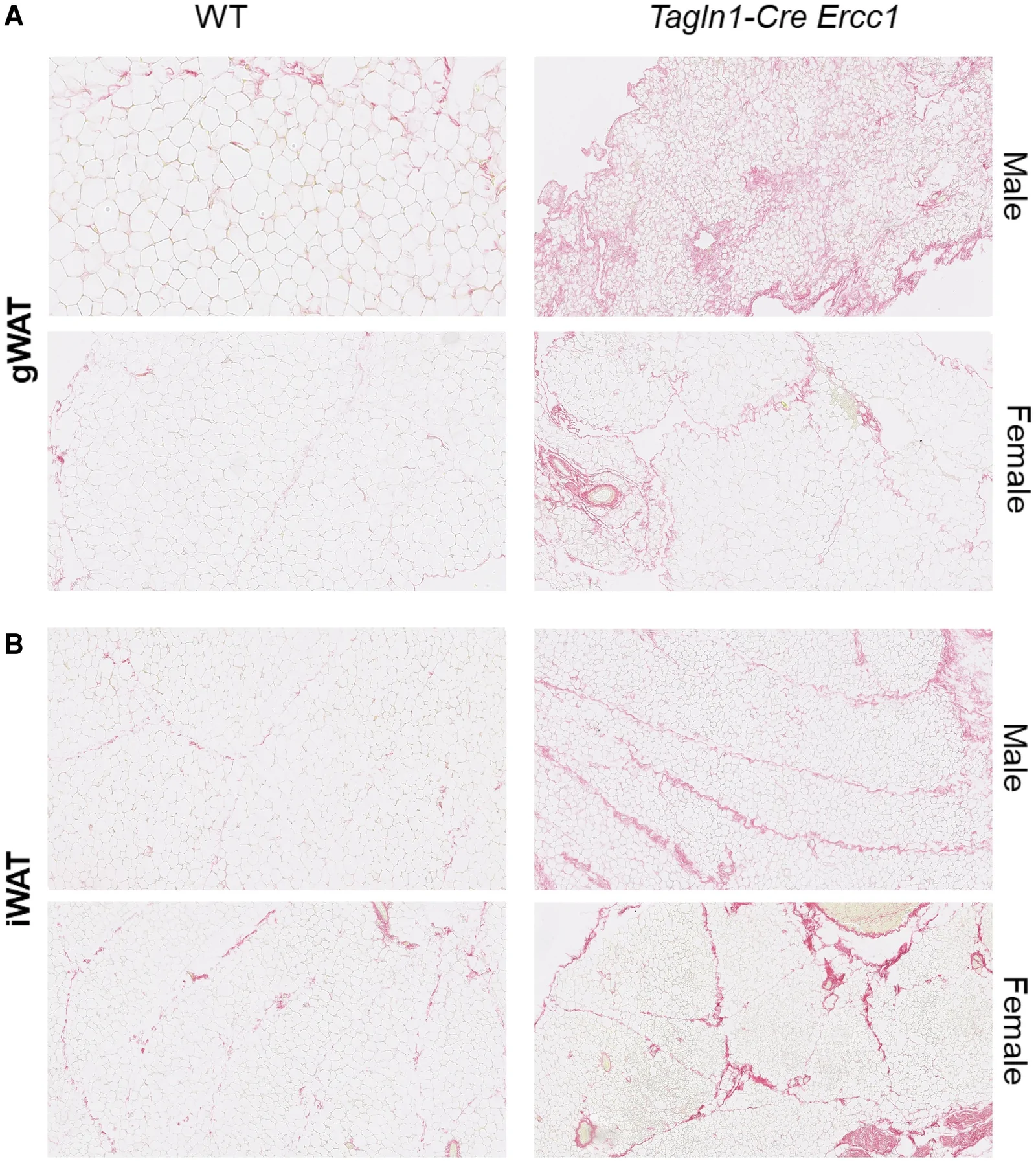
Morphological characterization of white adipose tissue (WAT) depots isolated from 22-week-old wild-type (WT) and *Tagln1-Cre* driven *Ercc1* knockout (KO; *Tagln1-Cre Ercc1*) mice. (A) Representative pictures of Picrosirius Red staining in gonadal WAT (gWAT). (B) Representative pictures of Picrosirius Red staining in inguinal WAT (iWAT).

Besides the age-related morphological changes of the adipocytes, we also observed remodeling of the microvasculature, marked by thickening of the vascular wall of small arteries, in gWAT and iWAT from male *Tagln1-Cre Ercc1* mice (39) (Fig. S1).

### Differences in mRNA expression in WAT from 8-week-old *Tagln1-Cre Ercc1* mice

Given the impact of *Tagln1-Cre*–driven Ercc1 deficiency on adipose tissue mass, we next analyzed the mRNA expression of adipokines and markers of glucose homeostasis, vascularization, aging, apoptosis, antioxidant, lipolysis, and lipogenesis per depot and sex at the age of 8 weeks.

There was no genotype effect on the expression of adipose tissue, glucose homeostasis, and apoptosis markers in all 3 WAT depots from male mice (Fig. 5, first and second panels). Furthermore, mRNA expression of vascularization markers was not different in gWAT and aWAT from male *Tagln1-Cre Ercc1* mice compared with male WT mice (Fig. 5, second panels). In contrast, in iWAT from male *Tagln1-Cre Ercc1* mice, endothelial nitric oxide synthase (*eNOS/Nos3*) mRNA expression was increased compared with WT mice (Fig. 5C, second panel).

**Figure 5:**
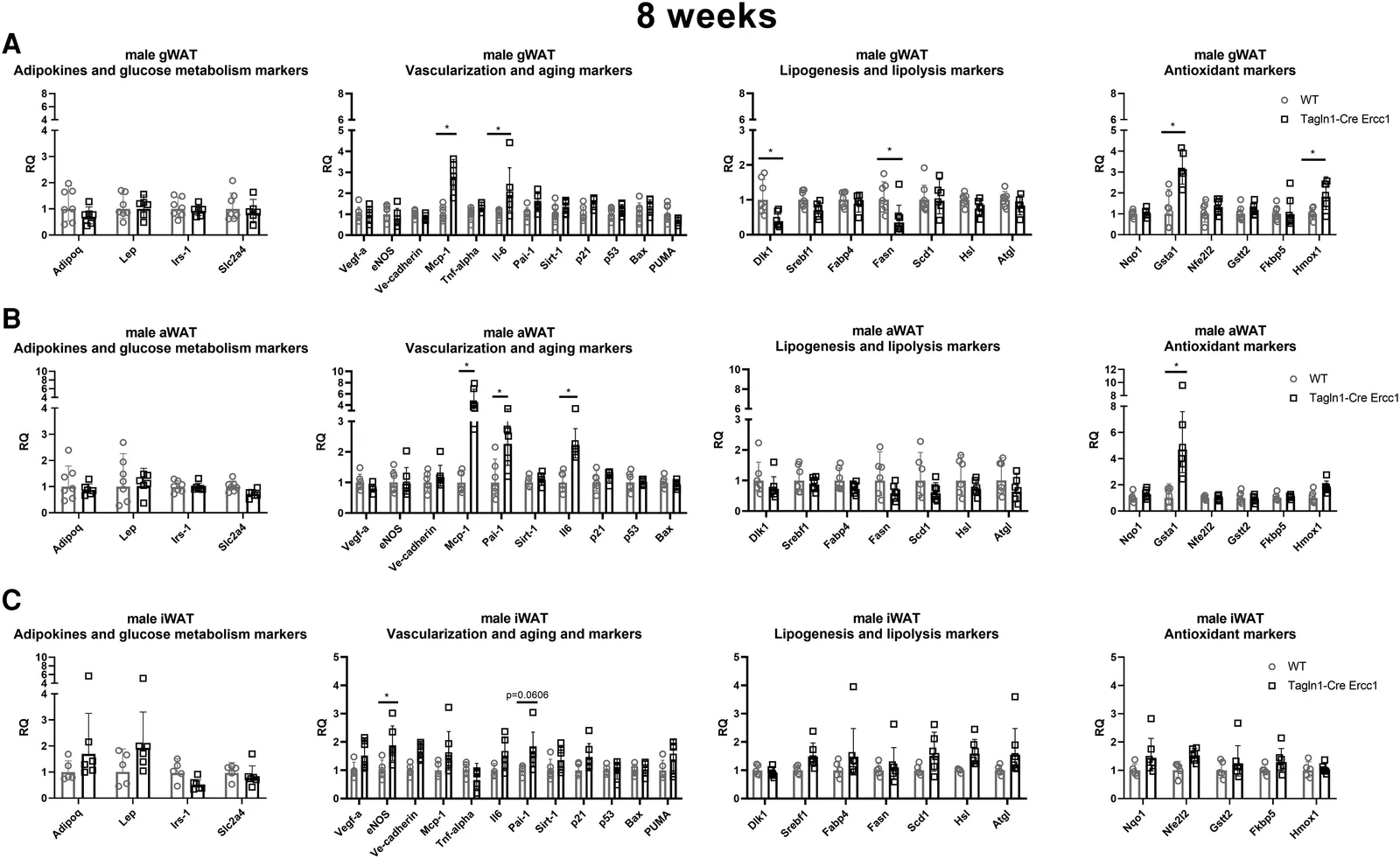
mRNA expression of adipose tissue markers, measured in white adipose tissue depots isolated from male 8-week-old wild-type (WT) and *Tagln1-Cre*–driven *Ercc1* knockout (KO; *Tagln1-Cre Ercc1*) mice. (A) gWAT, (B) aWAT, and (C) iWAT. Data were analyzed by 2-way ANOVA. *Significant effect of genotype with *P* < .05 by post hoc test corrected for the number of genes per gene panel. Abbreviations: aWAT, axillary white adipose tissue; gWAT, gonadal white adipose tissue; iWAT, inguinal white adipose tissue; RQ, relative quantity.

mRNA expression of inflammatory and aging markers *Mcp-1* and *Il-6* was increased in gWAT and aWAT from male *Tagln1-Cre Ercc1* mice compared with male WT mice (Fig. 5A and 5B, second panels). Moreover, *Pai-1* mRNA expression was increased in male aWAT from *Tagln1-Cre Ercc1* mice compared with WT mice, and there was a similar trend in iWAT without reaching statistical significance (*P* = .0606) (Fig. 5B and 5C, second panels).

The inhibitor of adipogenesis delta-like noncanonical Notch ligand 1 (*Dlk1*), which controls the fate of adipocyte progenitors (40), was downregulated in gWAT from male *Tagln1-Cre Ercc1* mice compared with male WT mice (Fig. 5A, third panel). Furthermore, mRNA expression of the lipogenesis marker fatty acid synthase (*Fasn*) was decreased in gWAT from male *Tagln1-Cre Ercc1* mice compared with male WT mice (Fig. 5A, third panel). There were no differences in other lipogenesis or lipolysis markers between male *Tagln1-Cre Ercc1* mice and male WT mice in the 3 analyzed WAT depots.

mRNA expression of the antioxidant marker glutathione-S-transferase α1 (*Gsta1*) was increased in gWAT and aWAT, while heme oxygenase 1 (*Hmox1*) levels were only increased in gWAT from male *Tagln1-Cre Ercc1* mice compared with male WT mice (Fig. 5, fourth panel). There were no changes in the other analyzed antioxidant markers across the 3 WAT depots from male mice (Fig. 5C, second, third, and fourth panels).

We confirmed decreased *Ercc1* mRNA expression in all male adipose tissue depots from *Tagln1-Cre Ercc1* mice compared with their corresponding WT mice (39) (Fig. S2A, left panel).

In gWAT from female *Tagln1-Cre Ercc1* mice, adiponectin (*Adipoq*) mRNA expression was increased compared with female WT mice (Fig. 6A, first panel). Furthermore, *Hmox1* mRNA expression was increased in aWAT from female *Tagln1-Cre Ercc1* mice compared with female WT mice (Fig. 6B, fourth panel). All other evaluated genes, except for *Mcp-1*, were not affected by the genotype in WAT from female mice (Fig. 6). *Mcp-1* mRNA expression was increased by ∼2-fold in the female WAT depots from *Tagln1-Cre Ercc1* compared with WT mice (Fig. 6).

**Figure 6:**
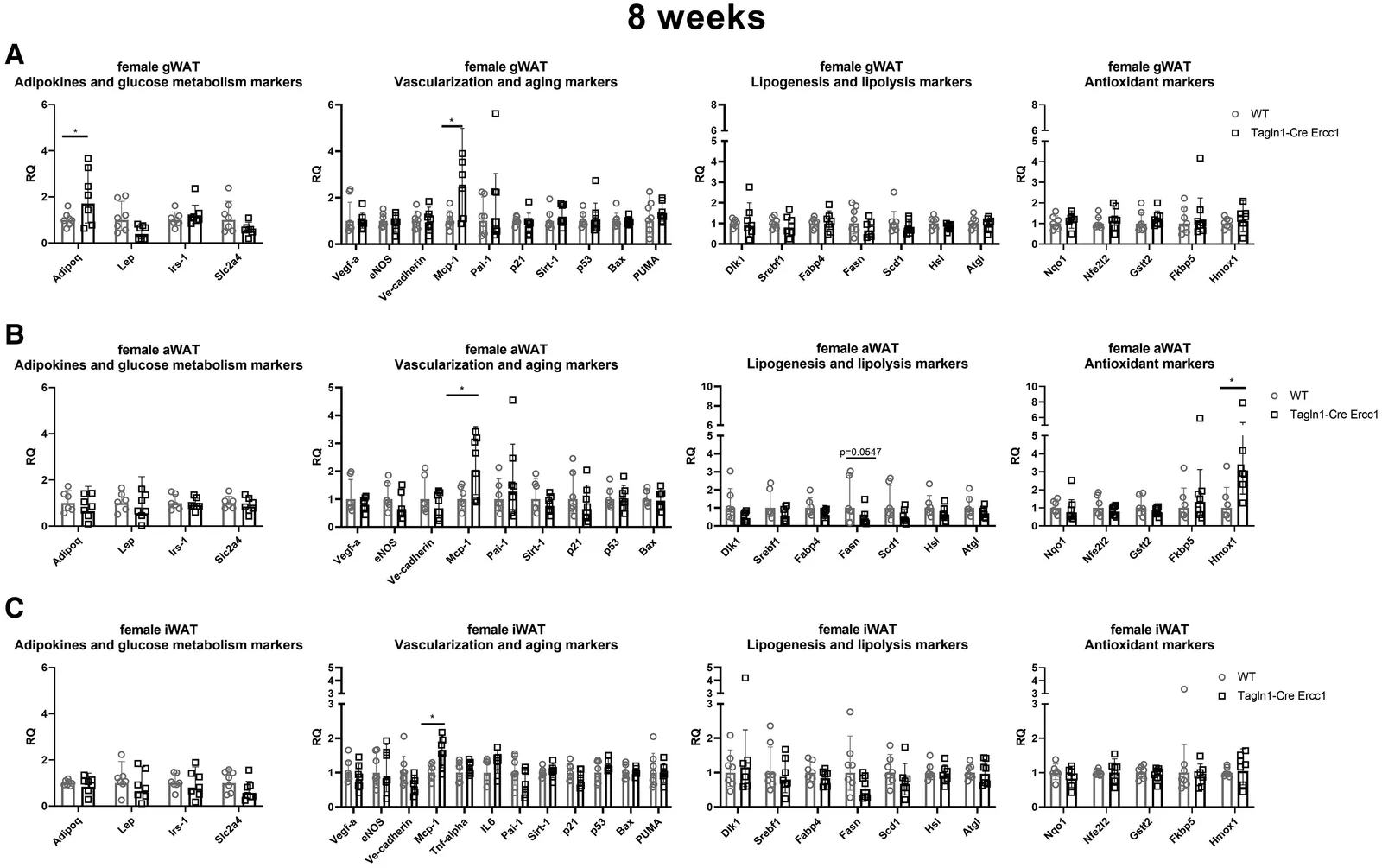
mRNA expression of adipose tissue markers, measured in white adipose tissue depots isolated from female 8-week-old wild-type (WT) and *Tagln1-Cre–*driven *Ercc1* knockout (KO; *Tagln1-Cre Ercc1*) mice. (A) gWAT, (B) aWAT, and (C) iWAT. Data were analyzed by 2-way ANOVA. *Significant effect of genotype with *P* < .05 by post hoc test corrected for the number of genes per gene panel. Abbreviations: aWAT, axillary white adipose tissue; gWAT, gonadal white adipose tissue; iWAT, inguinal white adipose tissue; RQ, relative quantity.

In the adipose tissue depots from 8-week-old female *Tagln1-Cre Ercc1* mice, *Ercc1* mRNA expression was only reduced in gWAT and iWAT compared with female WT mice, but not in aWAT (39) (Fig. S2A, right panel).

Upregulation of MCP-1 and F4/80, a marker for macrophage infiltration, is a typical feature of the chronic low-grade inflammation during aging (19, 20). Since gWAT secretes the majority of inflammatory markers compared with other adipose tissue depots (41), we performed immunohistochemical staining for F4/80 in gWAT from male and female *Tagln1-Cre Ercc1* and corresponding WT mice (Fig. 7, upper pictures). There were mild changes in the number of F4/80 positive cells between genotypes, with gWAT of both male and female *Tagln1-Cre Ercc1* mice having slightly more F4/80 positive cells compared with WT mice.

**Figure 7:**
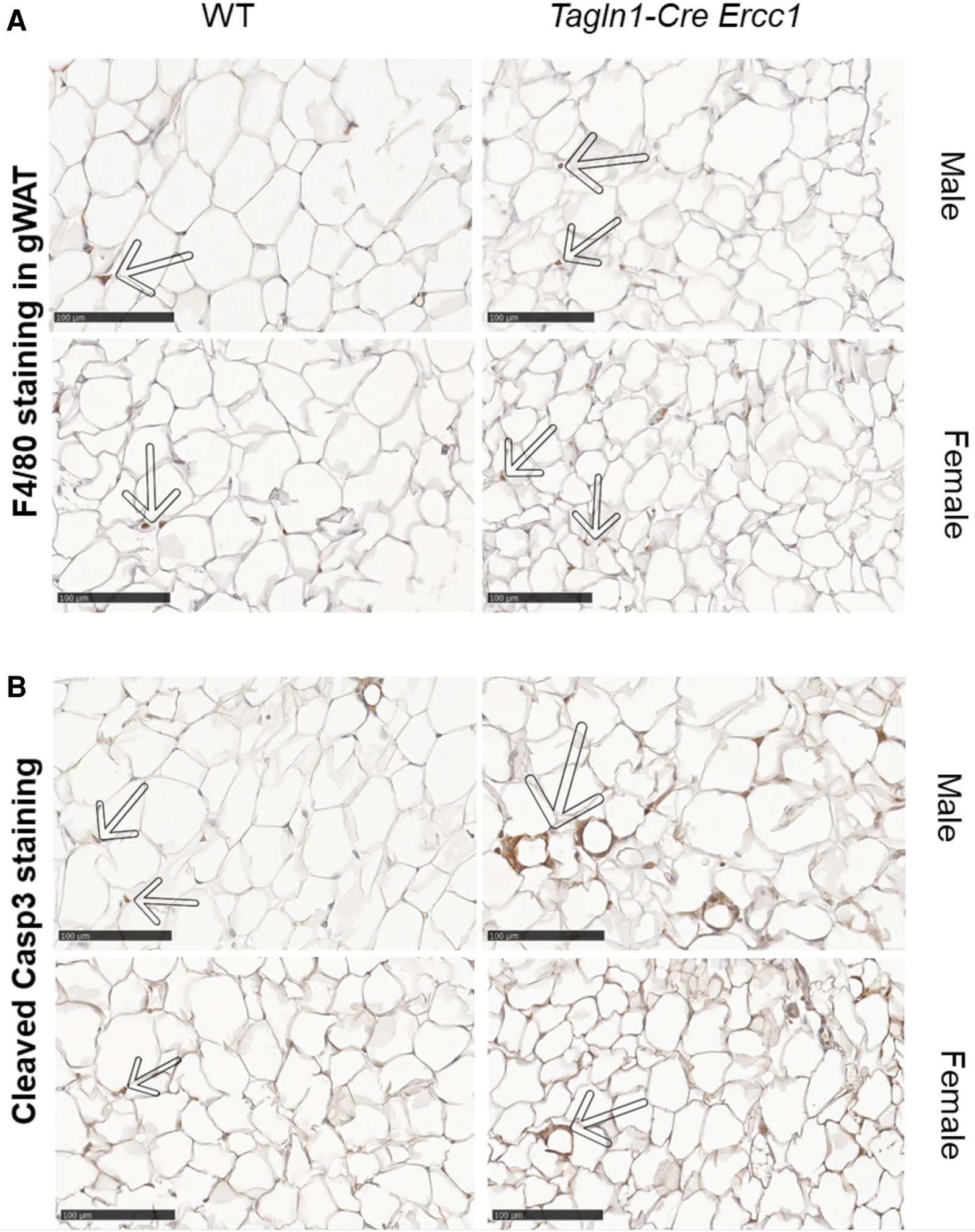
Immunohistochemical staining for macrophage infiltration (F4/80) (A) or apoptosis (cleaved caspase 3) (B) in white adipose tissue depots isolated from male and female 8-week-old wild-type (WT) and *Tagln1-Cre–*driven *Ercc1* knockout (KO; *Tagln1-Cre Ercc1*) mice. Arrows indicate positive staining.

Furthermore, we analyzed whether apoptosis played a role in the gWAT phenotype of *Tagln1-Cre Ercc1* mice by staining for cleaved caspase 3. There were more cleaved caspase 3–positive cells in male and female *Tagln1-Cre Ercc1* mice compared with male and female WT mice, respectively (Fig. 7, lower pictures). However, the overall genotype effects were subtle.

### Differences in mRNA expression in WAT from 22-week-old *Tagln1-Cre Ercc1* mice

At the age of 22 weeks, *Lep* was downregulated in gWAT depots from male *Tagln1-Cre Ercc1* mice compared with WT mice (Fig. 8A, first panel). Moreover, there was a trend of decreased *Mcp-1* and *Tnf-α* mRNA expression in male *Tagln1-Cre Ercc1* mice compared with male WT mice without reaching statistical significance (*P* = .0686 and *P* = .0757, respectively) (Fig. 8A, second panel). *P21* mRNA expression was significantly decreased in male *Tagln1-Cre Ercc1* mice compared with male WT mice. There was no genotype effect on any other evaluated markers in gWAT from male mice.

**Figure 8:**
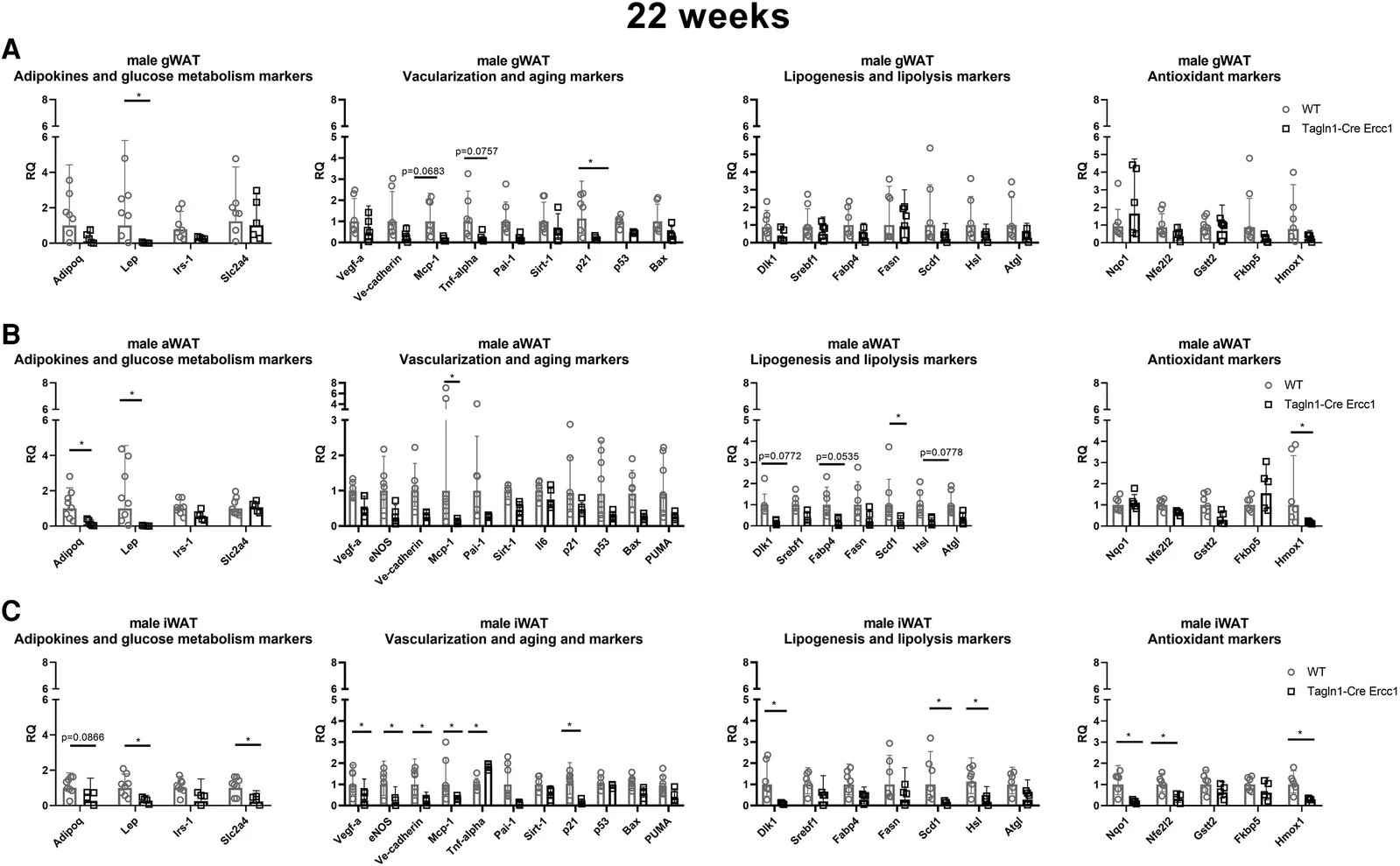
mRNA expression of adipose tissue markers, measured in white adipose tissue depots isolated from male 22-week-old wild-type (WT) and *Tagln1-Cre–*driven *Ercc1* knockout (KO; *Tagln1-Cre Ercc1*) mice. (A) gWAT, (B) aWAT, and (C) iWAT. Data were analyzed by 2-way ANOVA. *Significant effect of genotype with *P* < .05 by post hoc test corrected for the number of genes per gene panel. Abbreviations: aWAT, axillary white adipose tissue; gWAT, gonadal white adipose tissue; iWAT, inguinal white adipose tissue; RQ, relative quantity.

In male aWAT, *Adipoq* mRNA expression was downregulated in *Tagln1-Cre Ercc1* compared with male WT mice (Fig. 8B, first panel). Furthermore, *Mcp-1* mRNA expression was decreased in *Tagln1-Cre Ercc1* mice compared with WT mice (Fig. 8B, second panel). There were trends of decreased *Dlk1*, fatty acid binding protein 4 (*Fabp4*), and hormone-sensitive lipase (*Hsl*) mRNA expression in male *Tagln1-Cre Ercc1* mice compared with male WT mice without reaching statistical significance (*P* = .0772, *P* = .0535, and *P* = .0778, respectively) (Fig. 8B, third panel). Stearoyl-CoA desaturase-1 (*Scd1*) mRNA expression was significantly reduced in aWAT from male *Tagln1-Cre Ercc1* mice compared with WT mice. Furthermore, antioxidant marker *Hmox1* was decreased compared with male WT mice (Fig. 8B, fourth panel).

In male iWAT, there was a trend of decreased *Adipoq* mRNA expression without reaching statistical significance (*P* = .0866), and *Slc2a4* mRNA expression was significantly reduced in male *Tagln1-Cre Ercc1* mice compared with male WT mice (Fig. 8C, first panel). Furthermore, all analyzed vascularization markers were significantly decreased (Fig. 8C, second panel). *Mcp-1* and *P21* mRNA expression were reduced in male *Tagln1-Cre Ercc1* mice compared with male WT mice, while *Tnf-α* mRNA expression was slightly increased. There was no genotype effect for apoptosis markers in iWAT from male mice. *Dlk1*, *Scd1*, and *Hsl* mRNA expression were strongly reduced in iWAT from 22-week-old male *Tagln1-Cre Ercc1* mice compared with male WT mice (Fig. 8C, third panel). Furthermore, the mRNA expression of the antioxidant markers NAD(P)H quinone dehydrogenase 1 (*Nqo1*), nuclear factor, erythroid 2 like 2 (*Nfe2l2*), and *Hmox1* was decreased in male *Tagln1-Cre Ercc1* mice compared with male WT mice (Fig. 8C, fourth panel).

Moreover, we confirmed that *Ercc1* mRNA expression was reduced in all male WAT depots from *Tagln1-Cre Ercc1* mice compared with their corresponding WT littermates (39) (Fig. S2B, left panel).

In contrast, in female gWAT, there were no differences in adipokine and glucose metabolism markers between *Tagln1-Cre Ercc1* and WT mice (Fig. 9A, first panel). However, *Mcp-1* and *P21* mRNA expression were increased in female *Tagln1-Cre Ercc1* mice compared with female WT mice (Fig. 9A, second panel). No other markers were changed in female gWAT.

**Figure 9:**
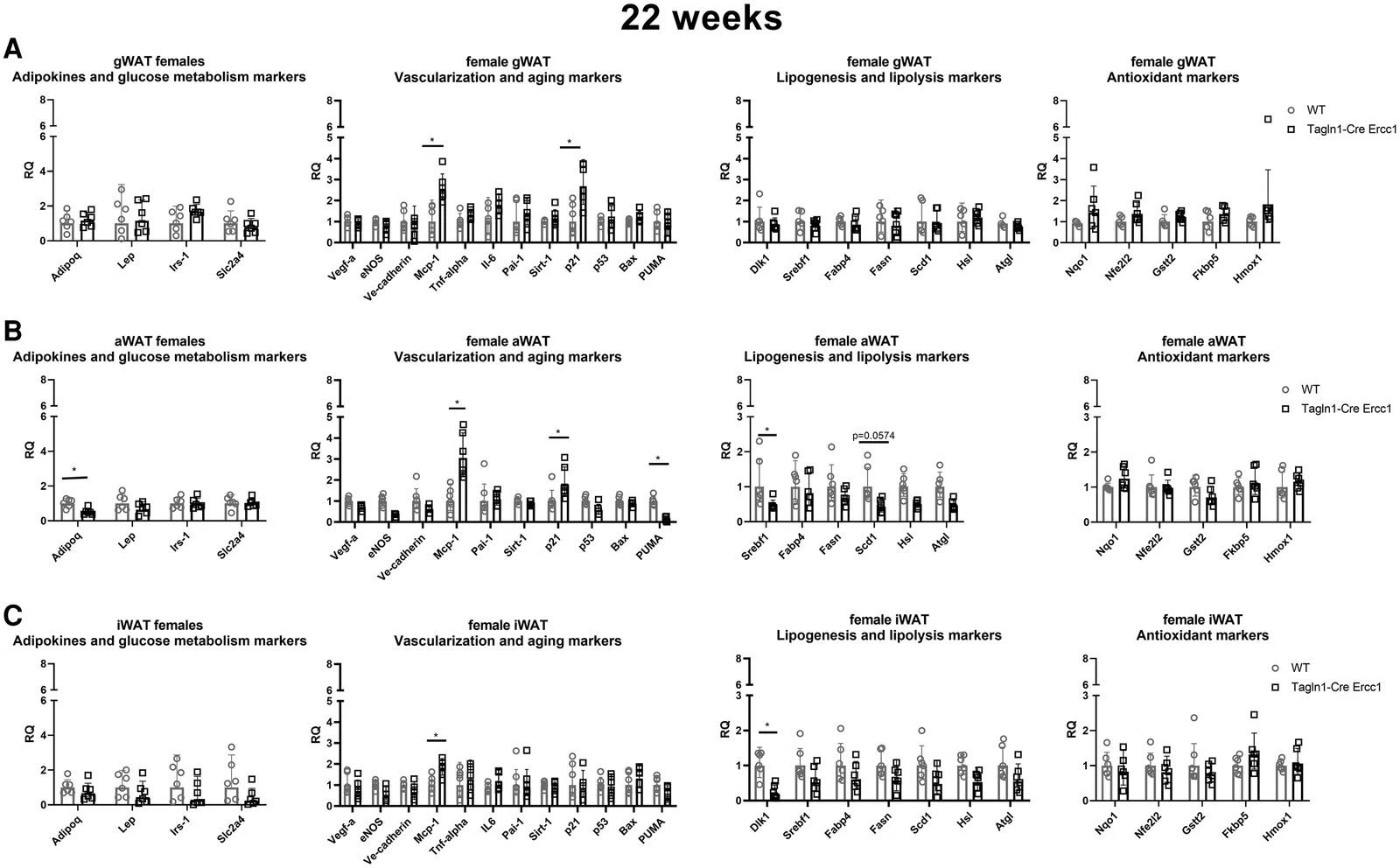
mRNA expression of adipose tissue markers, measured in white adipose tissue depots isolated from female 22-week-old wild-type (WT) and *Tagln1-Cre–*driven *Ercc1* knockout (KO; *Tagln1-Cre Ercc1*) mice. (A) gWAT, (B) aWAT, and (C) iWAT. Data were analyzed by 2-way ANOVA. *Significant effect of genotype with *P* < .05 by post hoc test corrected for the number of genes per gene panel. Abbreviations: aWAT, axillary white adipose tissue; gWAT, gonadal white adipose tissue; iWAT, inguinal white adipose tissue; RQ, relative quantity.

In female aWAT, *Adipoq* mRNA expression was decreased in *Tagln1-Cre Ercc1* mice compared with WT mice (Fig. 9B, first panel). Moreover, *Mcp-1* mRNA expression was increased, while the apoptosis marker p53 upregulated modulator of apoptosis (*Puma*) was decreased in female *Tagln1-Cre Ercc1* mice compared with female WT mice (Fig. 9B, second panel). There was no genotype effect on vascularization or antioxidant markers in female aWAT. mRNA expression of the lipogenesis marker sterol regulatory element-binding transcription factor 1 (*Srebf1*) was significantly reduced in female *Tagln1-Cre Ercc1* mice compared with female WT mice, and a similar trend was observed for the lipogenesis marker Scd1, which did not reach statistical significance (*P* = .0574) (Fig. 9B, third panel).

In female iWAT, there were only minor changes in mRNA expression, including increased *Mcp-1* and decreased *Dlk1* mRNA expression in *Tagln1-Cre Ercc1* mice compared with WT mice (Fig. 9C). All other analyzed markers were not affected by the genotype.

*Ercc1* mRNA expression was only reduced in aWAT and iWAT from 22-week-old female *Tagln1-Cre Ercc1* mice compared with female WT mice, but not in gWAT (39) (Fig. S2B, right panel).

Furthermore, we performed immunohistochemical staining for F4/80 and cleaved caspase 3 on gWAT sections from 22-week-old mice, where we observed more pronounced changes compared with gWAT from 8-week-old mice. In male and female WT mice, gWAT stained negative for F4/80. In contrast, we observed a high proportion of F4/80 positive cells in gWAT of *Tagln1-Cre Ercc1* mice, which appeared to be more pronounced in male mice compared with female *Tagln1-Cre Ercc1* mice (Fig. 10, top pictures). Moreover, we observed clusters of positive cells for cleaved caspase 3 in *Tagln1-Cre Ercc1* mice, which also appeared to be more pronounced in male mice compared with female *Tagln1-Cre Ercc1* mice (Fig. 10, bottom pictures). In male WT gWAT, there were some cells positive for cleaved caspase 3 staining; however, gWAT from female WT mice stained negative.

**Figure 10:**
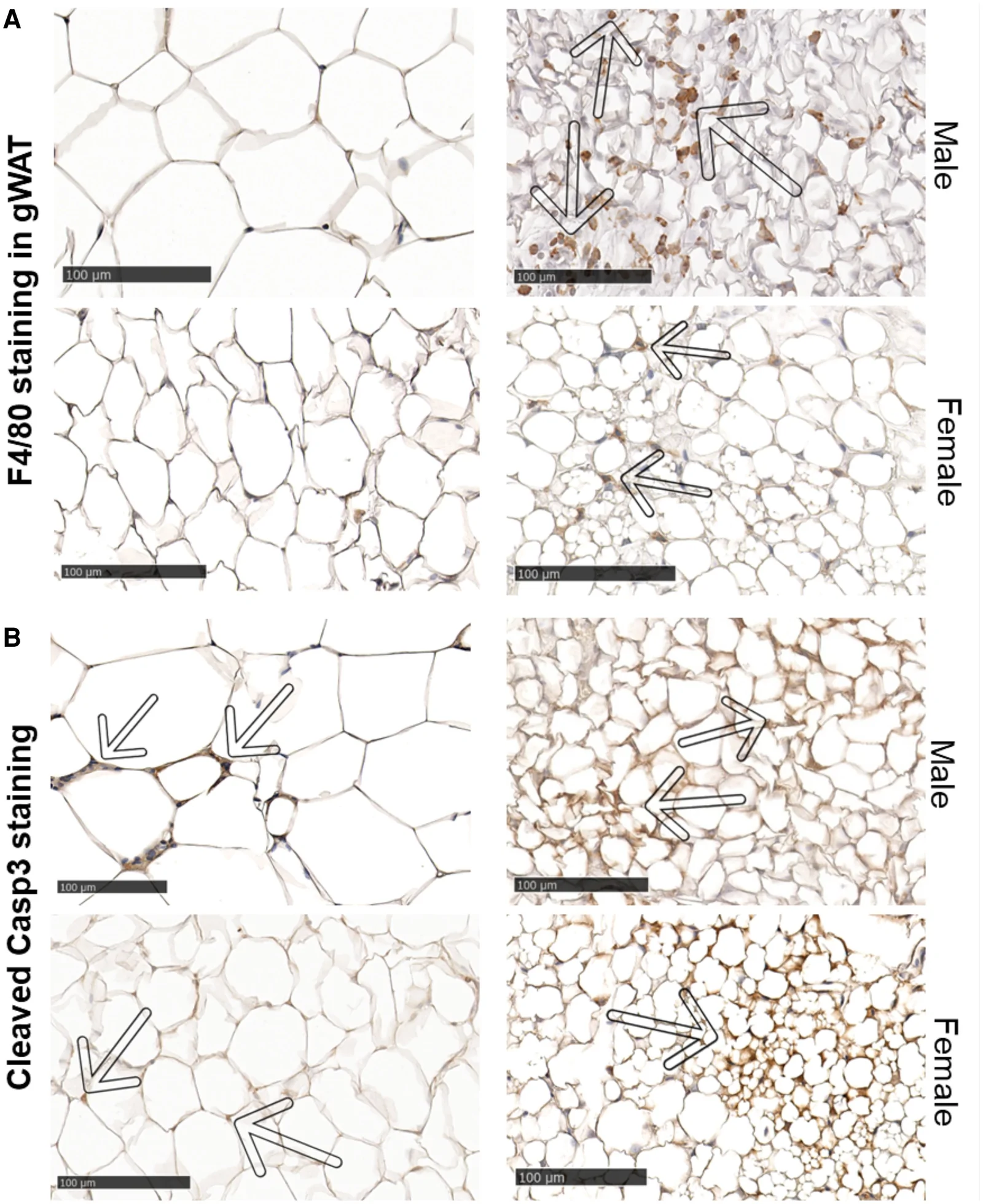
Immunohistochemical staining for macrophage infiltration (F4/80) (A) or apoptosis (cleaved caspase 3) (B) in white adipose tissue depots isolated from male and female 22-week-old wild-type (WT) and *Tagln1-Cre–*driven *Ercc1* knockout (KO; *Tagln1-Cre Ercc1*) mice. Positive staining is indicated by arrows.

### Effects of *Tagln1-Cre Ercc1* KO on BAT morphology and mRNA expression

Next, we evaluated changes in BAT at the age of 8 and 22 weeks (Fig. 11). At the age of 8 weeks, relative BAT weights and morphology were not significantly different between the genotypes and sexes (Fig. 11A). In contrast, at the age of 22 weeks, relative BAT weights were significantly lower in male *Tagln1-Cre Ercc1* mice compared with male WT mice, whereas there was no genotype effect in female mice (Fig. 11B, left panel). BAT of male *Tagln1-Cre Ercc1* mice had a denser appearance compared with male WT mice, which was confirmed by the decreased calculated lipid area in male *Tagln1-Cre Ercc1* mice compared with male WT mice (Fig. 11B, middle and right panels). In contrast, there were no morphological changes in female *Tagln1-Cre Ercc1* mice compared with female WT mice.

**Figure 11:**
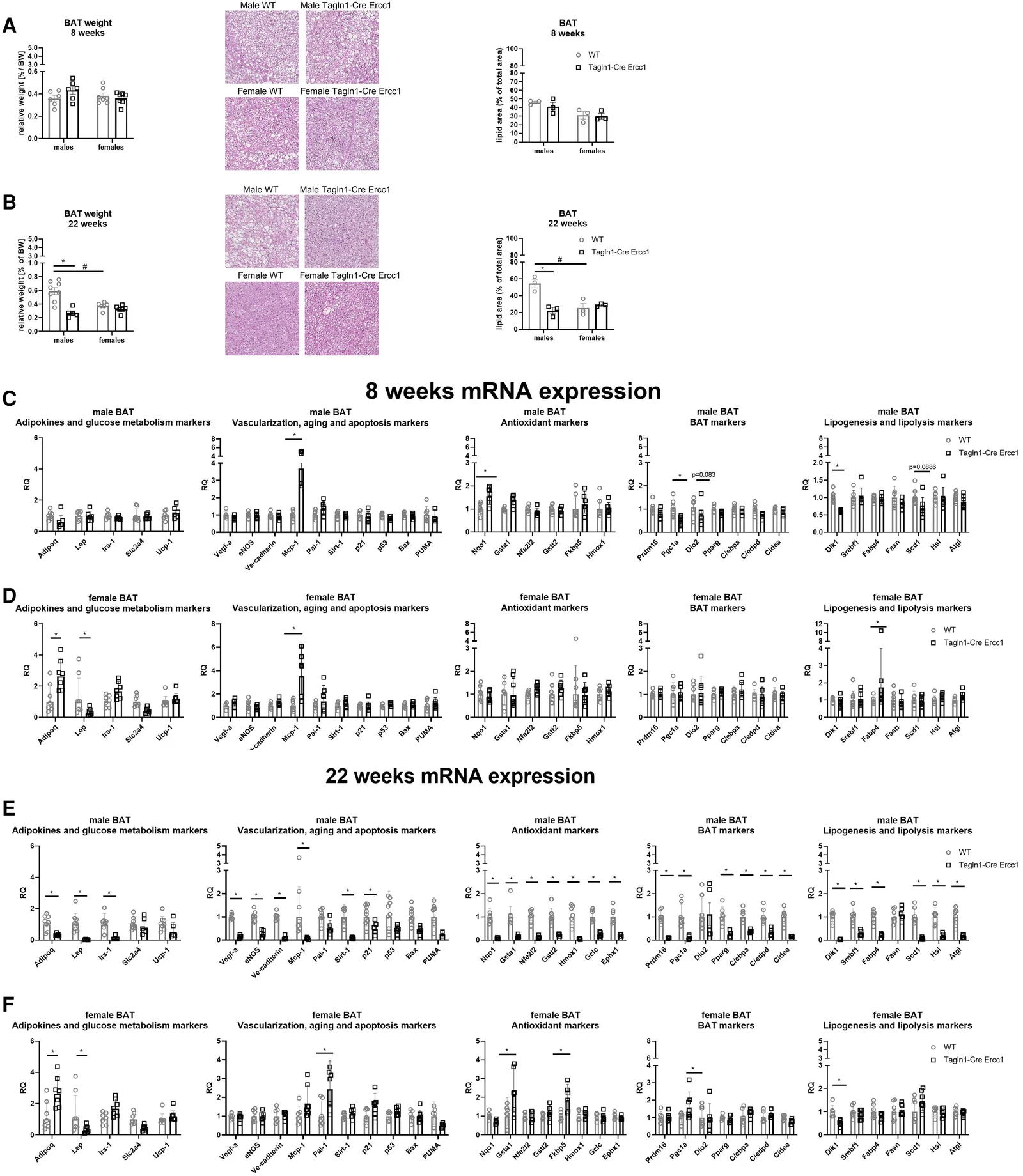
Characteristics of brown adipose tissue depot (BAT) isolated from wild-type (WT) and *Tagln1-Cre–*driven *Ercc1* knockout (KO; *Tagln1-Cre Ercc1*) mice. (A) Relative BAT weights and hematoxylin and eosin (H&E) staining pictures to assess morphological density, confirmed with lipid area calculation, respectively isolated from 8-week-old WT and *Tagln1-Cre Ercc1* mice. (B) Relative BAT weights and H&E staining pictures to assess morphological density, confirmed with lipid area calculation, respectively isolated from 22-week-old WT and *Tagln1-Cre Ercc1* mice. (C) mRNA expression of analyzed markers in BAT from male 8-week-old WT and *Tagln1-Cre Ercc1* mice. (D) mRNA expression of analyzed markers in BAT from female 8-week-old WT and *Tagln1-Cre Ercc1* mice. (E) mRNA expression of analyzed markers in BAT from male 22-week-old WT and *Tagln1-Cre Ercc1* mice. (F) mRNA expression of analyzed markers in BAT from female 22-week-old WT and *Tagln1-Cre Ercc1* mice. (A, B) There is a significant interaction of genotype × sex with *P* < .05 by 2-way ANOVA. ^#^Significant effect of sex. *Significant effect of genotype by post hoc test. (C-F) Analyzed with 2-way ANOVA. *Significant effect of genotype with *P* < .05 by post hoc test corrected for the number of genes per gene panel.

At the age of 8 weeks, there were only minor differences in mRNA expression in BAT from male *Tagln1-Cre Ercc1* mice, which included increased *Mcp-1* and *Nqo1* mRNA expression, and decreased peroxisome proliferator-activated receptor gamma coactivator 1-alpha (*Pgc1α*) and *Dlk1* mRNA expression compared with male WT mice. Moreover, a trend of reduced iodothyronine deiodinase 2 (*Dio2*) and *Scd1* mRNA expression was observed in *Tagln1-Cre Ercc1* mice without reaching statistical significance (*P* = .083 and *P* = .0885, respectively). The other evaluated genes were not affected by the genotype (Fig. 11C).

In contrast, in female BAT, *Adipoq* mRNA expression increased in 8-week-old *Tagln1-Cre Ercc1* mice, whereas *Lep* expression decreased compared with female WT mice (Fig. 11D, left panel). Furthermore, *Mcp-1* and *Fabp4* mRNA expression were increased in female *Tagln1-Cre Ercc1* mice compared with female WT mice, but no other evaluated genes were altered.

*Ercc1* mRNA expression was significantly decreased in BAT from 8-week-old male *Tagln1-Cre Ercc1* mice compared with male WT mice, while in females, there was no genotype effect (39) (Fig. S2A).

At the age of 22 weeks, *Adipoq*, *Lep*, and *Irs-1* mRNA expression were downregulated in BAT from male *Tagln1-Cre Ercc1* mice compared with male WT mice (Fig. 11E). Furthermore, all vascularization markers were strongly reduced in BAT from male *Tagln1-Cre Ercc1* mice compared with male WT mice, which was accompanied by decreased *Mcp-1*, sirtuin 1 (*Sirt-1*), and *P53* mRNA expression (Fig. 11E, second panel). The other evaluated aging and apoptosis markers were not different. Moreover, the mRNA expression of all evaluated antioxidant, lipolysis, lipogenesis (except for *Fasn*), and BAT markers (except for *Dio2*) was decreased in male *Tagln1-Cre Ercc1* mice compared with male WT mice (Fig. 11E, third to fifth panel).

In contrast to male *Tagln1-Cre Ercc1* mice, 22-week-old female *Tagln1-Cre Ercc1* mice showed a completely different molecular pattern in the BAT. *Pai-1* mRNA expression was significantly increased compared with female WT mice, while there was no genotype effect on vascularization markers, other aging and apoptosis markers (Fig. 11F, second panel). Furthermore, the mRNA expression of the antioxidant markers *Gsta1* and FK506-binding protein 51 (*Fkbp5*) were significantly increased in BAT from female *Tagln1-Cre Ercc1* mice compared with female WT mice, indicating a protective response (Fig. 11F, third panel). Correlating with this change, *Pgc1α* mRNA expression was significantly increased in female *Tagln1-Cre Ercc1* mice compared with female WT mice (Fig. 11F, fourth panel). *Dlk1* mRNA expression was decreased in *Tagln1-Cre Ercc1* mice compared with WT mice (Fig. 11F, fifth panel), while there was no genotype effect on other lipogenesis or lipolysis markers in female BAT.

*Ercc1* mRNA expression was strongly reduced in 22-week-old male BAT from *Tagln1-Cre Ercc1* mice compared with WT mice, while there was no genotype effect in female mice (39) (Fig. S2B).

### Adipose tissue depot–specific insulin sensitivity

Given the strong impact of *Tagln1-Cre*–driven Ercc1 deficiency on glucose tolerance in the male but not female mice, we performed an acute insulin test in male mice only. Assessment of phosphorylated AKT (pAKT) in adipose depots was used as readout of insulin signaling after injecting the mice with insulin or vehicle (saline) (Fig. 12A and corresponding Western blot pictures in Fig. 12B). In all adipose tissue depots of *Tagln1-Cre Ercc1* mice, insulin-induced pAKT levels were increased, but particularly for gWAT and BAT, Ercc1 deficiency led to increased insulin sensitivity compared with male WT mice (Fig. 12A, top-to-bottom panels, respectively). In the latter, insulin-induced pAKT levels only increased significantly in iWAT and aWAT. In gWAT and BAT, the higher insulin-induced pAKT levels did not reach statistical significance. The individual results of pAKT and total AKT for all depots can be found in the supplemental material (39) (Fig. S3).

**Figure 12:**
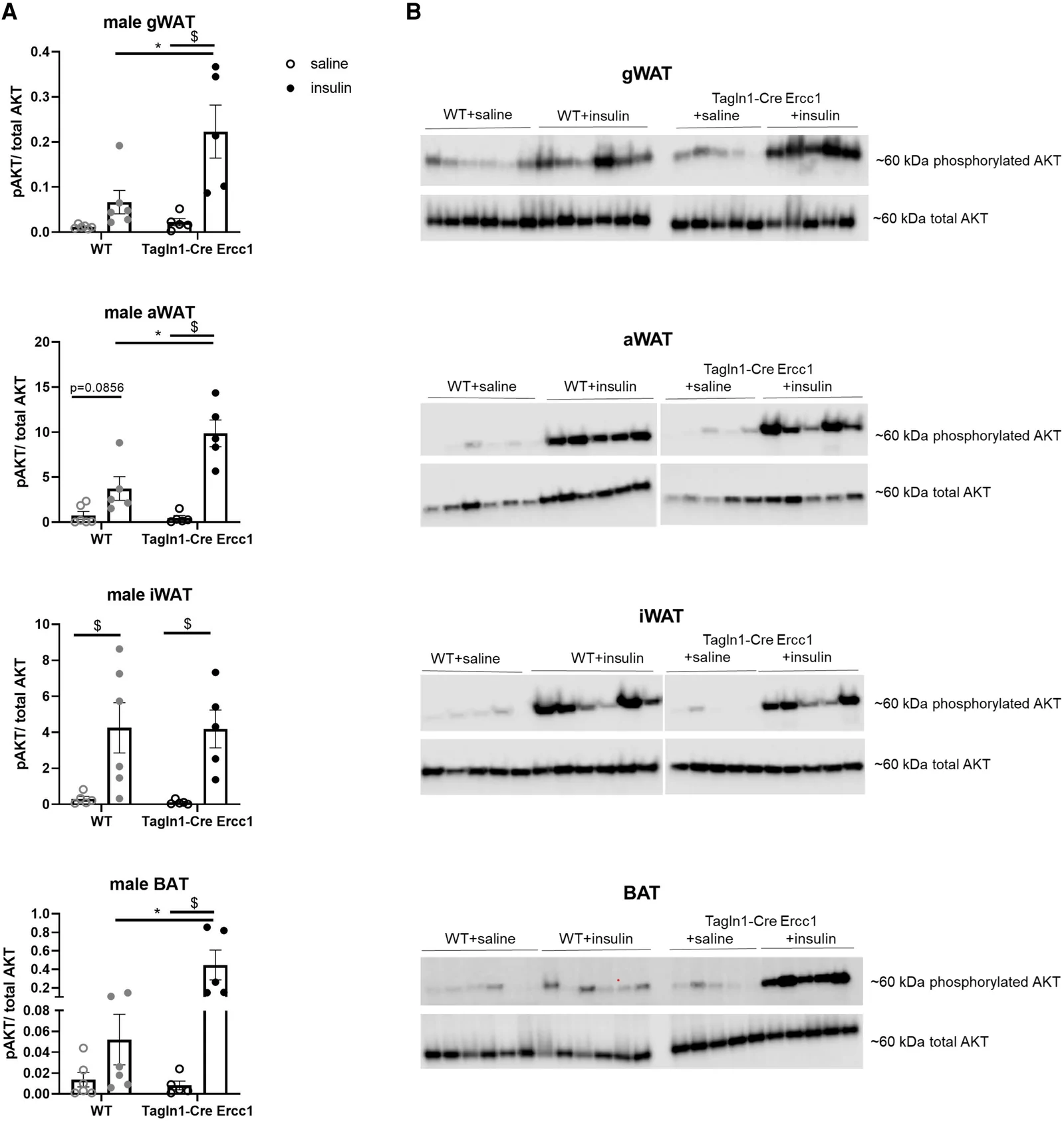
Insulin-stimulated AKT phosphorylation in adipose tissue depots. (A) Expression of total AKT and phosphorylated AKT (pAKT) in gonadal, subcutaneous, and inguinal WAT (gWAT, aWAT, and iWAT, respectively) and brown adipose tissue depots (BAT) after the acute insulin test performed in male mice. There is a significant interaction of saline/insulin injection × genotype with *P* < .05 by 2-way ANOVA. ^$^Significant effect of injection type. *Significant effect of genotype by post hoc test. (B) Corresponding Western blot pictures.

### Changes in the liver from *Tagln1-Cre Ercc1* mice

To investigate whether the changes in the adipose tissue depots in *Tagln1-Cre Ercc1* mice could be connected to age-related ectopic fat accumulation, we analyzed the H&E staining of liver tissue sections and measured the triglyceride content in liver homogenates from 8- and 22-week-old mice (Fig. 13A and 13B).

**Figure 13:**
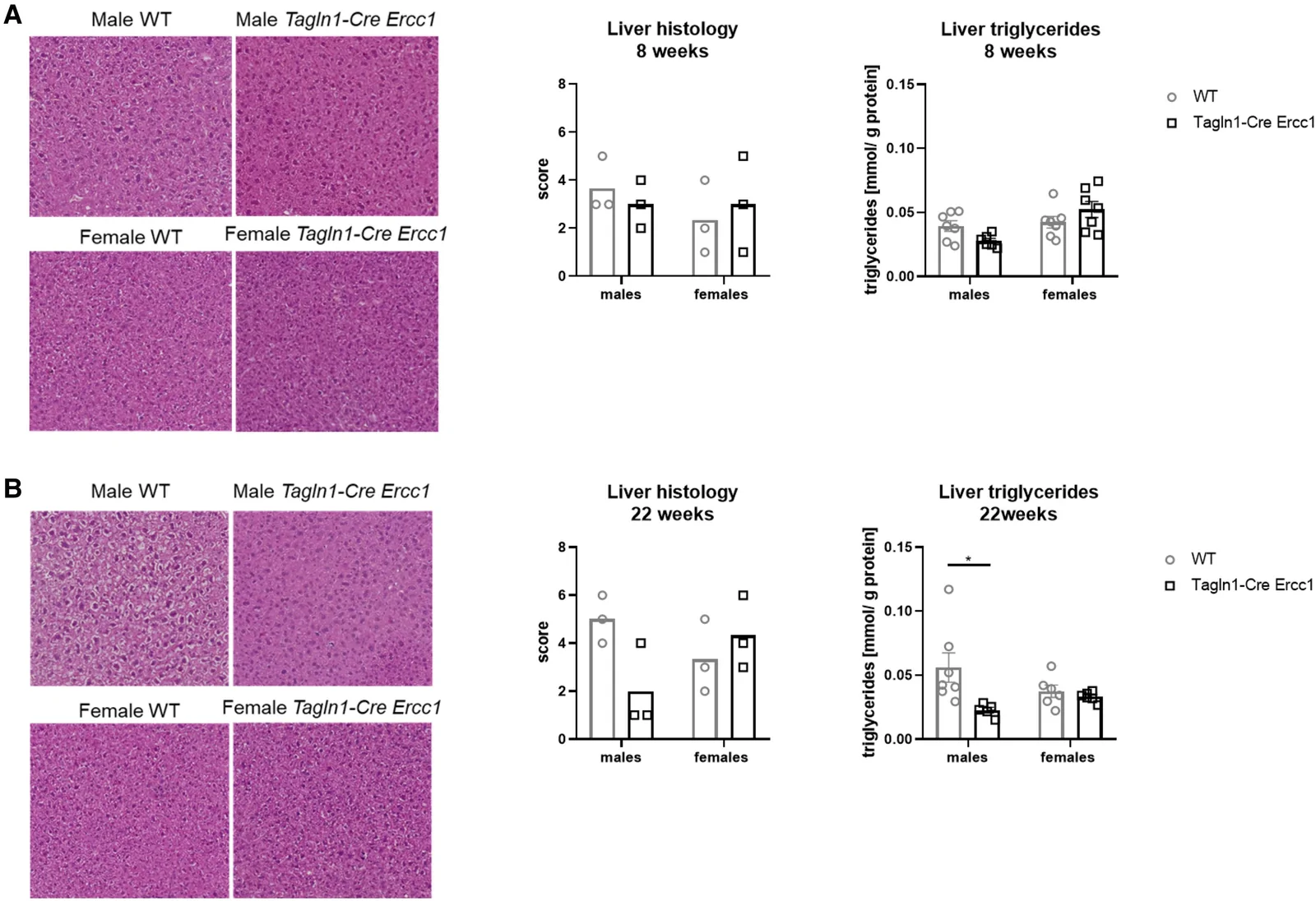
Lipids and triglycerides in the livers from wild-type (WT) and *Tagln1-Cre–*driven Ercc1 knockout (KO; *Tagln1-Cre Ercc1*) mice. (A) Hematoxylin and eosin (H&E) staining of the livers from 8-week-old WT and *Tagln1-Cre Ercc1* mice (left), corresponding scores of tissue compactness and lipids (middle) and liver triglycerides (right). (B) H&E staining of the livers from 22-week-old WT and *Tagln1-Cre Ercc1* mice (left), corresponding scoring of tissue compactness and lipids (middle) and liver triglycerides (right).

At the age of 8 weeks, there were no major differences in liver tissue compactness or number of lipids between *Tagln1-Cre Ercc1* and WT mice, independent of sex (Fig. 13A, left and middle panels). The triglyceride content in the livers from 8-week-old mice was also not different between the groups (Fig. 13A, right panel).

At the age of 22 weeks, there were more pronounced histological changes visible compared with the 8-week-old mice. Male *Tagln1-Cre Ercc1* mice showed a higher compactness of the liver tissue and less lipids compared with male WT mice (Fig. 13B, left and middle panels). In female mice, there was no genotype effect visible in the H&E staining. The triglyceride levels in the livers of male *Tagln1-Cre Ercc1* mice were reduced compared with male WT mice (Fig. 13B, right panel), but there was no genotype effect on triglyceride levels in female mice, which correlated with the histological assessment.

*Ercc1* mRNA expression was decreased in the livers from 8-week-old *Tagln1-Cre Ercc1* mice compared with WT mice, independent of sex. At the age of 22 weeks, there was a similar pattern, but in males, the reduction did not reach statistical significance (39) (Fig. S2C). To analyze whether in the skeletal muscle, another metabolically active tissue, *Ercc1* mRNA expression might also be affected, we performed similar measurements in skeletal muscle tissue at both ages. *Ercc1* mRNA expression was decreased in *Tagln1-Cre Ercc1* mice compared with WT mice, independent of sex and time point (39) (Fig. S2C).

As a positive control, we measured *Ercc1* mRNA expression in the lung, since this is an SMC-rich tissue. *Ercc1* mRNA expression was similarly decreased in lungs of both male and female *Tagln1-Cre Ercc1* mice compared with male and female WT mice, respectively, at the age of 22 weeks (39) (Fig. S1C).

### Potential role for transgelin in sex-dependent ERCC1 loss in the adipose tissue depots of *Tagln1-Cre Ercc1* mice

*Ercc1* KO in our mice was achieved using the *Tagln1-Cre* construct. Since, to our knowledge, sex-biased Cre activity is unknown, we analyzed whether sex differential expression of Tagln1 could have impacted our results. Therefore, *Tagln1* mRNA expression was measured in all adipose tissue depots from 22-week-old male and female WT mice. *Tagln1* mRNA expression differed sex- and depot-specifically (Fig. 14A). In gWAT and BAT, *Tagln1* mRNA expression was increased in female WT mice compared with male WT mice (Fig. 14A). However, in aWAT and iWAT, an opposite pattern was observed with decreased *Tagln1* mRNA expression in females compared with males. In the liver from female WT mice, *Tagln1* mRNA expression was increased 1.5-fold compared with male WT mice, and in skeletal muscle from female WT *Tagln1* mRNA expression increased 1.8-fold compared with male WT mice (*P* = .04 and *P* = .0002, respectively).

**Figure 14:**
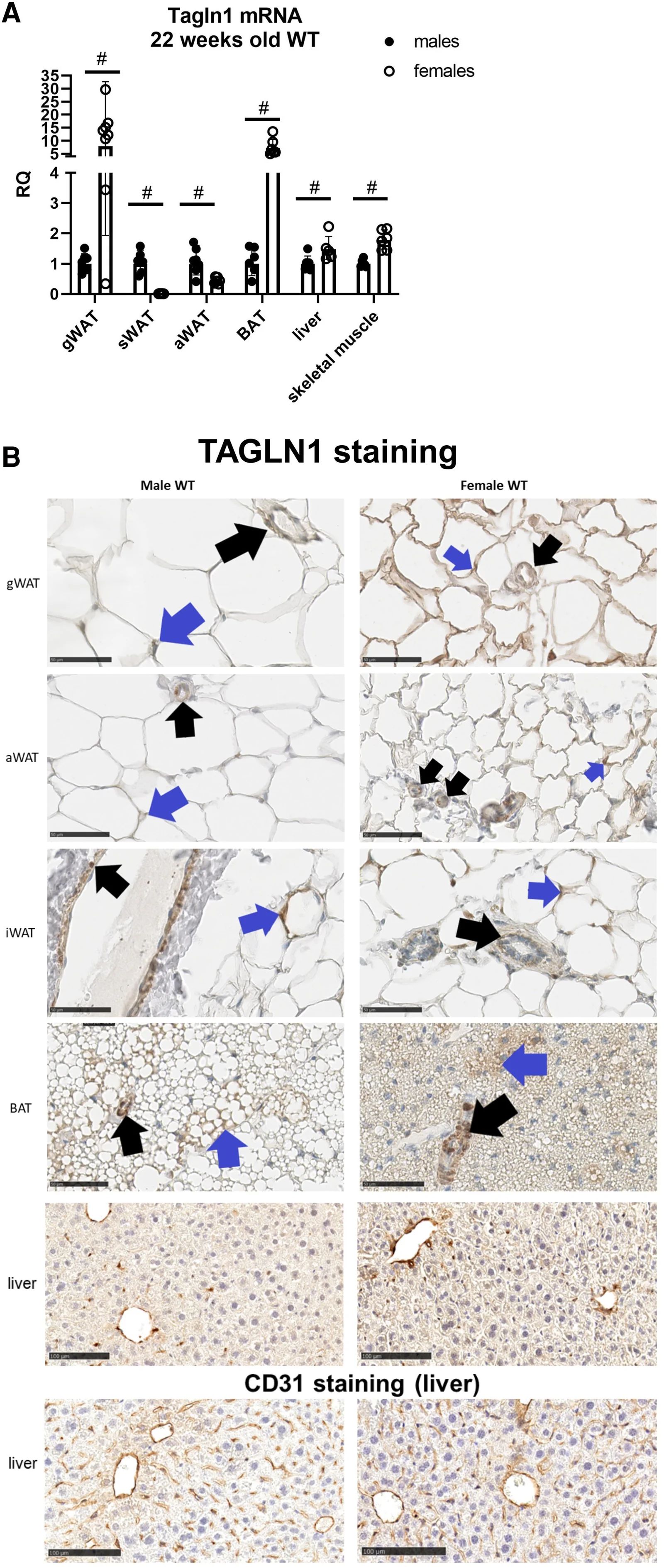
Tagln1 expression in adipose tissue depots and liver from 22-week-old wild-type (WT) mice. (A) *Tagln1* mRNA expression in adipose tissue depots from 22-week-old WT mice, analyzed with an unpaired *t* test. ^#^Significant effect of sex with *P* < .05. (B) Representative immunohistochemical staining for TAGLN1 in adipose tissue depots and in the liver from 22-week-old WT mice. Arrows in blue indicate positive staining for TAGLN1 around adipocytes, and black arrows indicate positive staining around arteries.

To investigate the localization of TAGLN1 within the different adipose tissue depots, we performed immunohistochemical staining on adipose tissue sections from 22-week-old WT mice (Fig. 14B). We observed a subpopulation of TAGLN1-positive adipocytes (marked by a blue arrow) in male and female gWAT, as well as in male and female aWAT and iWAT (Fig. 14B, left panels). Furthermore, in BAT we observed clusters of TAGLN1-positive adipocytes in male and female WT mice, which were generally more abundant in female BAT (Fig. 14B, left panels), which is in line with the mRNA data (Fig. 14A). Additionally, there was TAGLN1-positive staining in SMC in small arteries in all four adipose tissue depots (indicated with a black arrow).

We also analyzed the localization of Tagln1 in the liver given the reduced hepatic *Ercc1* mRNA expression. Only SMC in the (micro)vasculature of the liver expressed TAGLN1, and we confirmed the identity of small blood vessels with CD31 staining. We did not detect TAGLN1-positive staining in hepatocytes of male and female WT mice (Fig. 14B, bottom pictures).

To sum up, we observed sex- and adipose depot-specific differences in Tagln1 mRNA expression and protein localization, which possibly contributed to the observed sex differences in adipose depots of *Tagln1-Cre Ercc1* mice. Tagln1 expression appeared to be specific for SMCs and adipocytes in the analyzed tissues.

## Discussion

In this study, we evaluated the effect of *Tagln1-Cre*–driven *Ercc1* KO, a strategy used to induce DNA damage-based accelerated aging, on adipose tissue depot function and morphology and consequences for glucose homeostasis in mice. Male and female *Tagln1-Cre Ercc1* mice failed to gain adipose depot mass after 8 weeks of age. Additionally, male *Tagln1-Cre Ercc1* mice showed pronounced morphological and molecular changes in the adipose tissue depots, including smaller adipocytes, giving them a denser appearance compared with male WT mice. mRNA expression of vascularization markers was strongly decreased in all adipose tissue depots from male *Tagln1-Cre Ercc1* mice compared with male WT mice, which was accompanied by increased fibrosis and macrophage infiltration in WAT, suggesting low-grade inflammation typically occurring during aging. Moreover, male *Tagln1-Cre Ercc1* mice were more insulin sensitive compared with male WT mice, suggesting improved metabolic health. However, it is important to consider the obese-like phenotype of male WT mice, which has been reported previously (34) and partially contributed to the pronounced differences in the evaluated parameters compared with male *Tagln1-Cre Ercc1* mice. In contrast, in female *Tagln1-Cre Ercc1* mice, there were only minor morphological, molecular, and functional changes in the adipose tissue compared with female WT mice, despite the impairment of gaining fat mass over time. Thus, the consequences of ERCC1 deficiency in adipocytes and SMCs driven by *Tagln1-Cre* varied sex-specifically, with adipose tissue depots of male *Tagln1-Cre Ercc1* mice being more affected and female *Tagln1-Cre Ercc1* mice being partially protected. Moreover, we observed sex- and adipose tissue depot-specific *Tagln1* expression in WT mice, which is a novel finding for Tagln1.

The *Tagln1-Cre* promoter, which was used to drive *Ercc1* KO, is highly expressed in SMC-rich tissues like aorta, heart, and lung, whereas lower expression has also been reported in BAT (24, 42). Recently, Tagln1 has been reported to be a novel marker for a specific subpopulation of male white pre- and mature adipocytes (26). However, our study is the first to report that Tagln1 is expressed in a sex-specific manner in WAT and BAT of adult WT mice, which might contribute to differences in adipose tissue depot composition and function between male and female WT mice (43, 44). In male *Tagln1-Cre Ercc1* mice, ERCC1 deficiency occurred in all adipose tissue depots, leading to pronounced changes in morphology and function compared with male WT mice, while in female *Tagln1-Cre Ercc1* mice only partial ERCC1 deficiency was observed in the adipose tissue depots, correlating with mild morphological and functional changes compared with female WT mice. Vascular dysfunction has been observed in both male and female *Tagln1-Cre Ercc1* mice (16, 23), which correlated with a similar degree of ERCC1 deficiency in vascular (SMC-rich) tissue. Tagln1 is located on chromosome 11 (45), which is not a sex chromosome, and we currently know of no data that demonstrate sex-specific Ercc1 expression or Cre activity that could explain the sex dimorphic effects of Ercc1 KO in the adipose tissue of *Tagln1-Cre Ercc1* mice. Transgelin 2, a member of the same gene family as Tagln1, has a known sex-dimorphic function in adipose tissue (46), and in combination with our results, this suggests a sex-dependent role for both Tagln variants.

*Tagln1-Cre Ercc1* mice failed to gain adipose depot mass after 8 weeks of age, which was accompanied by pronounced morphological and molecular changes in male *Tagln1-Cre Ercc1* mice, including a decreased average adipocyte size, leading to a denser BAT and WAT morphology at the age of 22 weeks. Mean adipocyte size in aWAT and vWAT from male mice first increases with age (middle-aged mice) and then decreases with advanced age, accompanied by a reduction of total fat mass at the age of 24 to 30 months (12, 47, 48). Moreover, in mouse models of premature aging, which exhibit a severe phenotype in the adipose tissues, including loss of adipose tissue depot mass, average adipocyte size was significantly decreased compared with WT mice (19, 34, 48, 49). Our findings in male *Tagln1-Cre Ercc1* mice strongly resemble the adipose morphology of these advanced aged mice, suggesting an advanced aging phenotype in all adipose tissue depots in male *Tagln1-Cre Ercc1* mice. Impaired vascularization of the adipose tissue depots in advanced-aged male mice contributes to increased oxidative stress in WAT (48), which aligns with decreased vascularization and antioxidant markers in WAT from 22-week-old male *Tagln1-Cre Ercc1* mice compared with male WT mice. Moreover, the excessive accumulation of collagens during aging leads to adipose tissue fibrosis and thickening of the vascular wall of the microvasculature, contributing to impaired perfusion of the adipose tissue (48, 50, 51), which also aligns with our findings in male *Tagln1-Cre Ercc1* mice. Unfortunately, the studies discussed herein about advanced and premature aging mice did not include female mice. However, it is known that middle-aged female mice are partially protected from metabolic aging (10). Female *Tagln1-Cre Ercc1* mice showed smaller differences in adipose tissue morphology and gene expression compared with female WT mice and normal glucose tolerance despite the impairment in gaining fat mass. These minor changes could indicate a partial protection of female *Tagln1-Cre Ercc1* mice from metabolic aging persisting until advanced age. Indeed, the upregulation of antioxidant and mitochondrial markers in BAT from female *Tagln1-Cre Ercc1* mice compared with female WT mice suggests a protective response, which might have mitigated the metabolic phenotype in female Tagln1-Cre Ercc1 mice.

The impairment in gaining fat mass was not linked to increased lipolysis in the adipose tissue depots from *Tagln1-Cre Ercc1* mice. Furthermore, decreased mRNA expression of *Dlk1*, an inhibitor of adipocyte differentiation (40), in the adipose tissue depots from *Tagln1-Cre Ercc1* mice, suggests enhanced adipogenesis rather than compromised preadipocyte differentiation. However, further research is required to investigate potential alterations in preadipocyte differentiation in *Tagln1-Cre Ercc1* mice. Instead, the impairment to gain fat mass could potentially be linked to apoptosis in Tagln1-expressing preadipocytes. We indeed observed increased cleaved caspase 3 protein expression in gWAT from *Tagln1-Cre Ercc1* mice compared with WT mice starting at the age of 8 weeks and progressing until the age of 22 weeks. Senescent Ercc1-deficient fibroblasts secrete Tnf-α, which induces apoptosis in neighboring Ercc1-deficient fibroblasts (52). Similarly, Ercc1-deficient preadipocytes (white and brown) could undergo apoptosis, which could contribute to the hampered age-related fat mass gain in *Tagln1-Cre Ercc1* mice.

The increased IL-6 plasma levels in male *Tagln1-Cre Ercc1* mice suggest the presence of elevated systemic inflammation compared with male WT mice, which is also indicative of aging (13). Aging aWAT and epididymal adipose tissue have been identified as major sources of circulating proinflammatory cytokine IL-6 (41). At the age of 8 weeks, we observed increased mRNA expression of *Mcp-1* across all adipose tissue depots from *Tagln1-Cre Ercc1* mice, independent of sex, which was accompanied by increased *Il-6* mRNA expression compared with WT mice. Over time, this leads to chronic inflammation, which affects the composition and gene expression pattern of adipose tissue depots (1, 3) and might partially explain the downregulation of most of the evaluated markers in aWAT, gWAT, iWAT, and BAT from 22-week-old male *Tagln1-Cre Ercc1* mice compared with male WT mice. Moreover, MCP-1 is involved in the process of macrophage infiltration in WAT (53), which contributes to the progression of chronic low-grade inflammation during natural aging (19, 20) but has also been reported for premature aging (19), as observed in Hutchinson-Gilford progeria syndrome, which is marked by accelerated aging features in the whole body, loss of aWAT, and insulin resistance (54-57). In contrast to the insulin resistance observed in Hutchinson-Gilford progeria syndrome, insulin sensitivity improved in male *Tagln1-Cre Ercc1* mice and was unchanged in female *Tagln1-Cre Ercc1* mice compared with their WT counterparts. Systemic insulin sensitivity was also improved in naturally advanced-aged male mice and whole-body ERCC1-deficient male mice compared with young and corresponding male WT mice, respectively (12, 34), suggesting that male *Tagln1-Cre Ercc1* mice exhibit typical age-related metabolic changes. We excluded a contribution of ectopic lipid accumulation, which typically occurs with age (48) and is linked to impaired lipolysis driven by adipose tissue macrophages (58), to the metabolic phenotype of *Tagln1-Cre Ercc1* mice. Despite decreased lipolysis markers in WAT, male *Tagln1-Cre Ercc1* mice showed less lipids and triglycerides in the livers compared with male WT mice at the age of 22 weeks, suggesting enhanced metabolic health, which correlates with improved systemic insulin sensitivity in male *Tagln1-Cre Ercc1* mice compared with male WT mice. Furthermore, tissue-specific (BAT and WAT) insulin sensitivity was increased in male *Tagln1-Cre Ercc1* mice compared with male WT mice, suggesting that BAT and WAT functions are at least partially maintained. Preservation of function despite the low amount of fat mass was also observed in PVAT from *Tagln1-Cre Ercc1* mice (23), which mainly consists of beige adipose tissue depot, a mixture of BAT and WAT. Despite this partial preservation of function and denser appearance, the expression of most of the analyzed markers, including all BAT markers and the mitochondrial marker *Pgc1α* was decreased in BAT from male *Tagln1-Cre Ercc1* mice compared with male WT mice, which indicates impaired mitochondrial function and has been linked to disrupted BAT activity during aging (13, 59, 60). The lower BAT mass in male *Tagln1-Cre Ercc1* mice could potentially be linked to apoptosis in a subpopulation of preadipocytes, similar to what has been discussed for white preadipocytes above, which might also contribute to the reduced expression of BAT markers. Furthermore, it should also be taken into consideration that the large difference in fat mass between male *Tagln1-Cre Ercc1* and male WT mice could have contributed to the pronounced differences in the mRNA expression data, despite correction for cDNA (ng) and normalization to the housekeeping genes.

*Tagln1-Cre Ercc1* mice additionally displayed adaptations in energy metabolism, including decreased EE and increased RER compared with the corresponding WT mice. The reduced lean mass in *Tagln1-Cre Ercc1* mice could explain the decrease in EE. However, because EE is suppressed independently of lean mass (as assessed by analysis of covariance), there may be an additional effect of the genotype. The lower RER of male WT mice compared with male *Tagln1-Cre Ercc1* mice is possibly related to their increased fat mass and might be explained by shifts in energy metabolism toward utilizing fat as the primary fuel source instead of carbohydrates. Excess fat storage alters their metabolic pathways, encouraging fat oxidation and reducing reliance on carbohydrate metabolism (61). Additionally, the relative insulin resistance observed in the male WT mice compared with female mice suppresses carbohydrate metabolism, leaving the body to rely more on fat as a fuel source (62). The lower RER of male WT mice compared with male *Tagln1-Cre Ercc1* mice also correlates with decreased food intake in the light phase, which could be linked to their obese-like phenotype and has been reported for obese mice fed with a high-fat diet compared with lean mice (63). However, it cannot be ruled out that the altered feeding behavior in male *Tagln1-Cre Ercc1* mice is partly driven by changes in the hypothalamus, the main regulator of feeding and energy balance signals (64). However, *Tagln1* expression was not detected in single-cell expression data of the murine hypothalamus (65), suggesting that *Tagln1-Cre*–driven *Ercc1* deficiency probably does not play a major role in this brain region. Low levels of *Tagln1* have been reported in the pericytes in the blood–brain barrier (66). Therefore, *Tagln1-Cre–*driven *Ercc1* deficiency in pericytes could potentially impair blood-brain barrier function, similar to the observations in EC-specific *Ercc1* KO mice (67). Changes in blood–brain barrier function can alter the transport of metabolic signals, such as leptin, into the brain. Whether Tagln1 shows sex-differential expression in pericytes is unknown. Therefore, further research is warranted to unravel the mechanisms underlying the differences in food intake observed only between male *Tagln1-Cre Ercc1* mice and male WT mice.

One limitation of this study is that the obese-like phenotype of male WT mice, including relative insulin resistance compared with female WT mice, should be taken into consideration. However, similar observations regarding high body and fat mass and relative insulin resistance have previously been reported for male WT mice with an FVB::C57b6/J hybrid background (34), which suggests that the hybrid strain background also contributes to these metabolic sex differences. Across strains, female mice are generally more insulin sensitive compared with male mice (68), and females also show a milder phenotype to high-fat diet and genetically induced forms of insulin resistance compared with male mice (69, 70). Therefore, the FVB::C57b6/J hybrid strain background could also induce a milder metabolic effect in female WT mice compared with male WT mice, which could partially explain the observed sex differences between the WT mice. As a consequence, the observed sex differences between the WT mice are more pronounced.

Furthermore, decreased *Ercc1* mRNA expression in the liver and skeletal muscle (bulk tissue) might also affect glucose homeostasis in male *Tagln1-Cre Ercc1* mice. Since TAGLN1 was only present in VSMCs and not in hepatocytes, we concluded that using Tagln1 as the promoter to drive *Ercc1* KO, VSMCs are most likely the only cells in the liver with ERCC1 deficiency. Whether this loss of Ercc1 expression hampers hepatocyte function remains to be studied. Given that despite decreased *Ercc1* mRNA expression in the liver and skeletal muscle, female *Tagln1-Cre Ercc1* mice did not show changes in glucose homeostasis compared with female WT mice, the contribution of possible altered liver and muscle function to the metabolic phenotype in *Tagln1-Cre Ercc1* mice is likely minor.

To conclude, we showed that genomic instability induced in VSMC and Tagln1-dependent adipocyte populations leads to an acceleration of WAT and BAT aging and remodeling, decreased vascularization, and increased inflammation, which is also observed in normal aging. Male and female *Tagln1-Cre Ercc1* mice failed to gain fat mass over time, with males being more prominently affected compared with females. This phenotype included partial preservation of adipose tissue function and insulin sensitivity in *Tagln1-Cre Ercc1* mice. Moreover, we discovered sex- and depot-specific differences in Tagln1 expression, which could indicate a sex-dimorphic role of Tagln1 that might have contributed to the sex differences in *Tagln1-Cre Ercc1* mice. Our results contribute to improving the understanding of adipose tissue aging related to genomic instability in the context of specific tissue development drivers such as Tagln1 and consequences thereof for glucose homeostasis.

## Data Availability

All original data generated and analyzed during this study are included in this article or in the data repositories listed in the References.
